# Phytomechanochemical engineering of Bi/MgO interfaces for enhanced solar hydrogen production and multicomponent antibiotic degradation

**DOI:** 10.1039/d6ra08280g

**Published:** 2026-11-09

**Authors:** Ihsan Ullah, Muqaddas Kiran, Minhas Nazir, Merfat Alsabban, Khloud Al-Thubaiti, Amal Altowerqi, Francis Verpoort, Muhammad Babar Taj, Ismat Bibi, Rajeh Alotaibi

**Affiliations:** a Institute of Chemistry, The Islamia University of Bahawalpur Bahawalpur 63100 Pakistan dr.taj@iub.edu.pk; b Department of Chemistry, University of Jeddah Jeddah 21589 Saudi Arabia; c Department of Chemistry, King Abdulaziz University Jeddah 21589 Saudi Arabia; d Department of Physics, University of Taibah Madinah 42353 Saudi Arabia; e State Key Laboratory of Advanced Technology for Materials Synthesis and Processing, Wuhan University of Technology Wuhan 430070 China; f Department of Chemistry, College of Science, King Saud University Riyadh 11451 Saudi Arabia raalotaibi@ksu.edu.sa

## Abstract

The development of multifunctional photocatalysts that produce renewable hydrogen (H_2_) while removing persistent pharmaceutical pollutants offers a promising solution for sustainable energy and water remediation. Herein, we report the first green phytomechanochemical strategy for synthesizing Bi-loaded MgO (Bi_*x*_@MgO; *x* = 0.2, 0.5, or 0.7 wt% MgO) photocatalysts using clove (*Syzygium aromaticum*) extract as a natural reducing/stabilizing agent combined with solvent-free mechanochemical engineering. Unlike traditional chemical methods for preparing Bi-modified MgO, the current method combines phytochemical processes of Bi^3+^ reduction and a mechanochemical process for dispersing Bi onto MgO, forming the Bi/MgO interface without post-synthesis annealing. Bi addition leads to modification of both optical and electronic properties of MgO with a simultaneous increase of defect-mediated charge transfer. Bi_0.7_@MgO showed a decreased band gap of 2.78 eV and more efficient charge separation than that of pure MgO. Under optimal conditions Bi_0.7_@MgO exhibited a hydrogen production rate of 33.44 mmol g^−1^ h^−1^ when illuminated with solar light. The same photocatalyst also removed 98–99% of amoxicillin, cefixime, azithromycin, and ciprofloxacin under simulated solar light irradiation while maintaining high recyclability over six consecutive cycles.

## Abstract 2

Green phytomechanochemical engineering of Bi@MgO enables efficient solar hydrogen production and simultaneous degradation of hazardous antibiotics through enhanced charge separation and oxygen-vacancy-mediated photocatalysis.

## Graphical abstract

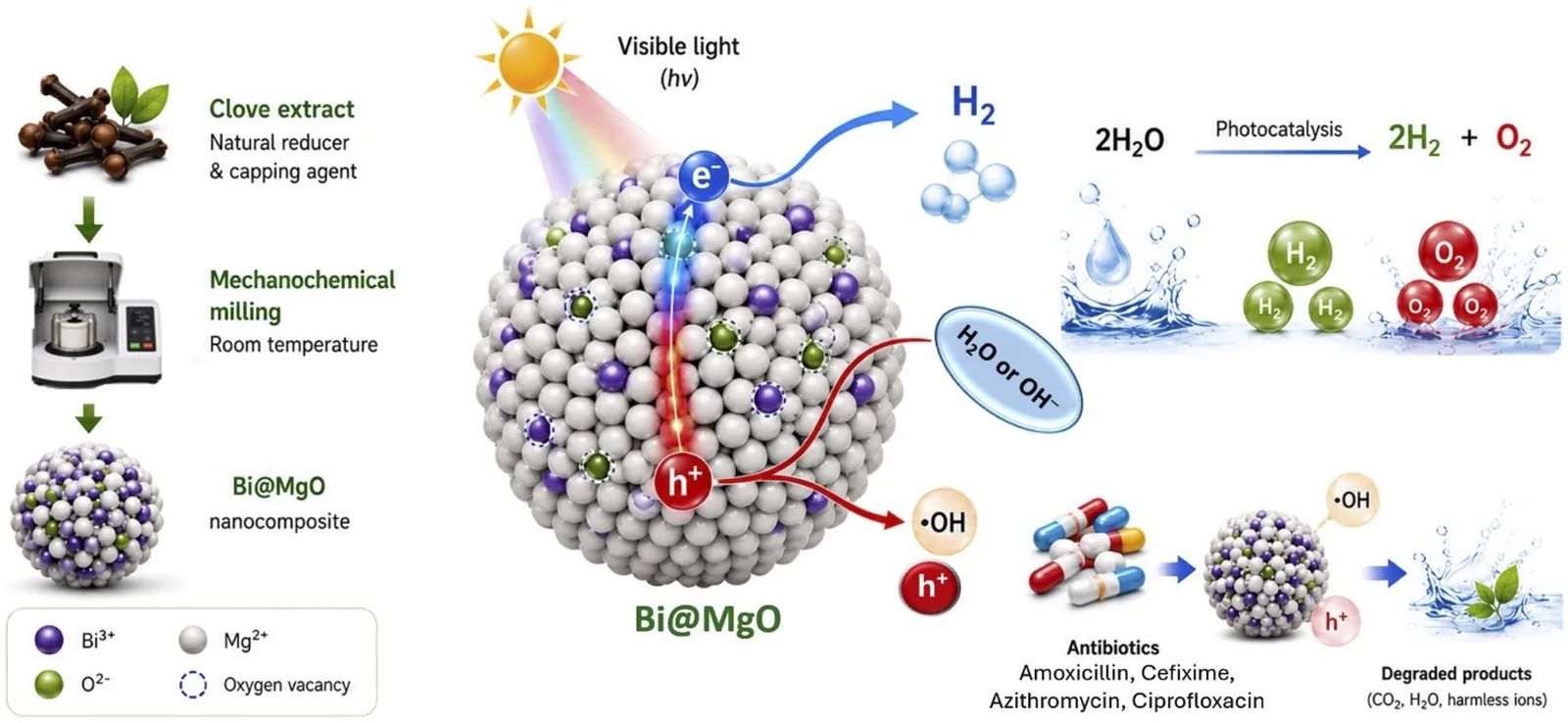

## 1 Introduction

Today's society is faced with two of the biggest issues that we must address: rapidly decreasing fossil fuel reserves and an increasing global energy demand, along with increasing environmental pollution.^1^ Thus, one possible source of clean energy is H_2_ and it has several features that make it appealing. H_2_ has a high gravimetric energy density (142 MJ kg^−1^), produces no carbon dioxide when combusted, and can be used in fuel cells.^2^ Recent research has focused on the production of H_2_ using photocatalysis, whereby solar energy is directly converted to chemical energy in the form of hydrogen by using semiconductor materials under mild operating conditions.^3^ The lack of available technology for large-scale H_2_ production using photocatalysis is due to several factors hindering the performance of photocatalysts; these include low solar-light utilization, low kinetics of water-splitting reactions, and the rapid recombination of charge carriers (electrons and vacancies) generated during the photocatalytic process.^4^ As a result of these factors, the research objective for photocatalytic H_2_ production is to develop relatively low-cost, environmentally friendly, highly efficient, and stable photocatalysts for H_2_ production *via* photocatalysis to enable large-scale H_2_ production.

The rise of emerging contaminants in water systems has become one of the greatest global concerns.^5^ Pharmaceutical residues, especially antibiotics, are routinely found in municipal and industrial wastewater because conventional biological treatment processes do not completely mineralize these compounds.^6^ By continuously discharging antibiotics into natural water bodies, the threats posed to aquatic ecosystems are exacerbated; moreover, there is the promotion of antimicrobial resistant microorganisms leading to significant public health threats.^7^ Advanced oxidation processes based on semiconductor photocatalysis have been shown a significant potential for permanently degrading persistent pharmaceutical pollutants by creating highly reactive oxidative species, therefore providing a sustainable alternative for purifying wastewater.^8^ Thus, the development of multifunctional photocatalysts capable of providing renewable energy generation and cleaning the environment is one of the most promising pathways toward fulfilling sustainable development goals.

Of many semiconductor photocatalysts, magnesium oxide (MgO) has gained significant interest because of its high chemical stability, low toxicity, abundant resources on earth, low price; as well as being a good base for surface reactions (having many active sites on their surface).^9^ Because of these features, magnesium oxide would be an excellent material to use when reacting both with water and with organic pollutants using photocatalysts. However, pure MgO so far only has a bandgap of about 5 eV or greater, resulting in its response mainly in the ultraviolet portion of the solar spectrum, which accounts for only about 4% of sunlight (by weight).^10^ A comparison of the literature reveals that pristine MgO usually has limited photocatalytic capability for H_2_ production. For instance, porous MgO crystals were able to yield around 320 µmol g^−1^ h^−1^ H_2_ from methanol through UV light over a long period, with the activity linked to the structure of MgO and its surface electronic properties. Similarly, MgO/g-C_3_N_4_ heterostructures have been shown to achieve a concentration of 30.1 µmol h^−1^ under visible light irradiation with the high activity resulting from the charge separation happening in the heterojunctions formed. Thus, it may be concluded from previous studies (SI: Table S1) that MgO is capable of participating in H_2_ production process under photocatalytic conditions; however, its photocatalytic efficiency can be noticeably improved if specific morphology, heterojunction formation, or other catalytic components are present. As such, improvements to the electronic structure must be made to MgO in order to improve both the ability to absorb visible light as well as the ability of different charges within the MgO material to separate from one another (for use in photocatalysts).^11^

Several tactics have been used in the past to enhance the photocatalytic characteristics of MgO, including creating heterojunctions, defect engineering, loading with metals, and loading surfaces.^12^ Of these procedures, adding Bi is one of the most beneficial because it has a unique electronic configuration (6s^2^ electrons that make up lone pairs). Bismuth (Bi) has a high degree of polarizability and good electron interactions with metal oxide semiconductors, making it an ideal candidate for this application.^13,14^

The concept of loading Bi has been studied as an approach to modifying the electrical and optical characteristics of photocatalysts that are based on magnesium oxide. Bi incorporation can add various electronic states as well as change the characteristics of charge transfer at the interface, which will affect the absorption of light and the dynamics of the charge carriers. However, the photocatalytic characteristics of the Bi–MgO systems depend heavily not only on the amount of Bi but also on its chemical state, its spatial distribution, and the surrounding area. Therefore, the synthetic route by which Bi is introduced can be of great importance for the characteristics of the resulting defect structure and electronic properties.^13,15^

Most previously reported Bi-modified MgO materials have been synthesized by conventional solution-based techniques such as co-precipitation/chemical precipitation and related wet chemical methods.^14^ These methods violate the principles of sustainable and green chemistry. In contrast, mechanochemical synthesis has been established as an environmentally friendly, industrially scalable method for producing nanomaterials.^16–21^ The use of phytochemicals in mechanochemistry opens new prospects for the use of plant molecules as reducing, complexing, or stabilizing agents instead of using synthetic additives.

In the present work, clove (*Syzygium aromaticum*) extract was selected as phytochemical ingredient for reducing Bi^3+^ into Bi metal and creating Bi/MgO interfaces. The main assumption of the work is based on the idea of combining phytochemical and solid-state mechanochemistry for regulating the features of Bi-doped MgO. Therefore, a series of Bi_*x*_@MgO materials with varying concentrations of Bi (0.2, 0.5, and 0.7 wt%) were prepared, and systematically characterized to elucidate their structural, optical, surface, and photoelectrochemical properties.

The photocatalytic performance of the synthesized materials was subsequently evaluated for simulated solar-driven H_2_ production, followed by the degradation of selected antibiotics such as amoxicillin (AMX), cefixime (CFX), azithromycin (AZM), and ciprofloxacin (CIP) using optimized photocatalyst. Together, these two photocatalytic processes provided a unified mechanism for charge transfer and generation of the reactive species and explanation of structure–property–performance relations in Bi_*x*_@MgO for photocatalytic applications. Consequently, the innovation of this work resides principally in the use of the phytochemical-assisted mechanochemical synthesis method and its ability to generate a photocatalytically active Bi/MgO interfacial system, rather than in the first preparation of Bi-modified MgO itself.

## 2 Materials and methods

### 2.1 Materials

Bismuth chloride, BiCl_3_ (≥99%), and magnesium hydroxide, Mg(OH)_2_ (≥98%) in the synthesis of pure and Bi_*x*_@MgO NPs. Precursors were purchased from Merck (Darmstadt, Germany). Double-distilled water was used as a preparation medium. The cloves (*Syzygium aromaticum*) buds were purchased from a local herbal store.

### 2.2 Preparation of pure and Bi_*x*_@MgO NPs

#### 2.2.1 Mechanochemical synthesis of MgO

Magnesium oxide (MgO) nanoparticles were produced using a solvent-free mechanochemical method followed by thermal annealing. 5 g of Mg(OH)_2_ was placed into a 50 mL zirconia jar with zirconia balls (10 balls, each having a 10 mm diameter, the weight ratio (ball to powder) weight ratio 20 to 1). The jar was placed in a single station planetary ball mill at 500 rpm for 3 hours (10 minutes off after every 30 minutes to avoid excess heat generation). After going through mechanical processing, the Mg(OH)_2_ particle size is decreased, the lattice strain is increased, and more structural defects have formed. The resultant milled powder was then taken and put into an alumina crucible and heat-treated in an ambient air muffle furnace at 500 °C for 2 h, with a heating rate of 5 °C min^−1^. During heat treatment, the Mg(OH)_2_ undergoes complete dihydroxylation as shown by eqn (1):

1 Mg(OH)_2_ → MgO + H_2_O

After naturally cooling to room temperature, the obtained white MgO powder was gently ground using an agate mortar and pestle and stored in an airtight container for further characterization and composite preparation.

#### 2.2.2 Preparation of clove extract and mechanochemical synthesis of metallic bismuth (Bi^0^)

##### 2.2.2.1 Preparation of clove extract

Freshly dried clove buds (*Syzygium aromaticum*) purchased from a local herbal supply, cleaned with de-ionized water to remove impurities on the surfaces, and then dried in an oven at 60 °C for 10 hours. The dried cloves were ground very finely with a mortar and pestle. 10 g of the clove powder was added to 200 mL of ethanol/water (30 : 70) and then heated to 50 °C under constant stirring. The resulting suspension was then cooled to room temperature and passed through Whatman No. 1 filter paper. The resulting filtrate was then converted into a concentrated extract by evaporating all of the solvent using a rotary evaporator (45 °C; reduced pressure). The remaining concentrated extract was dried overnight in a vacuum oven at 50 °C, producing a dark brown solid extract that was also ground into a fine powder and stored in a desiccator until use.

##### 2.2.2.2 Mechanochemical synthesis of Bi^0^

Metallic Bi NPs were obtained by using a mechanochemical reduction process without solvents. BiCl_3_ (3.15 g or 10 mmol) and the dried clove extract powder (1.5 g) were mixed and milled in a single station planetary ball mill (specification described above) for 4 hours (10 minutes of pause after every 30 minutes to control the temperature). The polyphenolic constituents of the clove extract acted as natural reducing agents (promoted the reduction of Bi^3+^ to metallic Bi^0^) and stabilizers for the Bi^0^. The product was cooled to room temperature and was directly used for the preparation of Bi_*x*_@MgO composites without any additional thermal treatment.

#### 2.2.3 Mechanochemical synthesis of Bi_*x*_@MgO (Bi_0.2_@MgO, Bi_0.5_@MgO, and Bi_0.7_@MgO)

Three Bi_*x*_@MgO composites containing nominal Bi^0^ weight percentages of 0.2, 0.5, or 0.7 wt%, hereafter designated as Bi_0.2_@MgO; Bi_0.5_@MgO; Bi_0.7_@MgO, respectively, were synthesized by a secondary mechanochemical process. The quantified amounts of the Bi NPs and 2.0 g of MgO were subjected to a single station planetary ball mill (the conditions mentioned in Bi^0^ synthesis) for 1 hour (10 minutes of pause after 30 minutes to control the temperature). The repetitive mechanochemical collision results in homogeneous dispersion of metallic Bi^0^ across the external surface of MgO, also increasing the interfacial contact area between both materials. Upon completion of milling, the composite powders were allowed to cool naturally to room temperature, gently homogenized using an agate mortar, and stored in airtight glass vials for subsequent physicochemical characterization and photocatalytic evaluation. No post-synthesis thermal treatment or annealing was applied.

### 2.3 Characterization of pure and Bi_*x*_@MgO NPs

All techniques used for analyzing the as-synthesized catalysts, including instrument names and specifications, are discussed in the SI.

### 2.4 Hydrogen production experiment

Photocatalytic H_2_ generation was carried out in a Pyrex photoreactor (140 mL, UK) under simulated solar irradiation (with little modification in the reported protocol).^22^ For each experiment, 5 mg of photocatalyst (optimized amount) was dispersed in 25 mL of a reaction mixture containing methanol and water (5 : 95 v/v). Before exposure to simulated solar light (the incident irradiance at the reactor surface was adjusted to 1000 W m^−2^ using a calibrated spectroradiometer (ILT970-VNIR Spectroradiometric Measurement System, International Light Technologies, Inc., Peabody, Massachusetts, USA) under irradiation from a 300 W xenon (Xe) lamp equipped with a 420 nm band-pass filter), the suspension was purged with argon to remove dissolved oxygen and headspace gases. H_2_ was monitored by withdrawing 0.5 mL headspace gas samples at regular intervals and analyzing them using a gas chromatograph (GC) (Tianmei GC7900, with N_2_ as the carrier). Quantification of H_2_ was performed using an internal calibration curve. Each photocatalytic test was repeated three times for reproducibility. The hydrogen production rate was calculated in mmol g^−1^ and mmol g^−1^ h^−1^ for comparison across MgO, Bi_0.2_@MgO, Bi_0.5_@MgO, and Bi_0.7_@MgO photocatalysts. All reactions were conducted for 6 h using 5% methanol as a sacrificial agent. The apparent quantum yield (AQY) was calculated *via*eqn (2).

2 AQY (%) = (*R*_H_2__ × 2× *c* × *h*/*I* × *λ* × *A*) × *N*_A_ × 100

where *R*_H_2__ is the rate of H_2_ produced (mol s^−1^), *c* is the speed of light (2.99 × 10^8^ m s^−1^), *h* is the Plank's constant (6.62 × 10^−34^ J s^−1^), *I* is the light intensity (1000 W m^−2^), *λ* is the wavelength of incident light (420 nm), *A* is the illuminated area of reactor (m^2^), and *N*_A_ is the Avogadro's constant (6.022 × 10^23^ mol^−1^).

### 2.5 Photocatalytic degradation of antibiotics

The effectiveness of photocatalytic degradation of pristine MgO with Bi_0_._7_@MgO was tested using AMX, CFX, AZM, and CIP as target antibiotics. The experiments were performed in a photoreactor at room temperature and pressure. The impact of various experimental variables was assessed to determine the optimal degradation conditions for antibiotics. The photocatalyst was mixed with the antibiotic solution and kept stirring in dark for 20 minutes in order to reach adsorption–desorption equilibrium before the start of irradiation process. Under visible light irradiation, the mixture was continuously stirred and 3 mL of liquid was drawn after every 20 minutes to monitor the degradation process. The degradation efficiency was calculated according to eqn (3):

3 Degradation (%) = [*C*_0_ − *C*_*t*_/*C*_0_] × 100

where *C*_0_ represents the TC concentration at the beginning of irradiation, following dark adsorption–desorption equilibration, and *C*_*t*_ represents the residual TC concentration after irradiation for time *t*.

## 3 Results and discussion

### 3.1 Fourier transform infrared (FTIR) analysis

In FTIR spectra Fig. 1a of Bi_0.2_@MgO, Bi_0.5_@MgO, and Bi_0.7_@MgO, a clear trend shows the effect of increased Bi^0^ concentration.^23^ In Bi_0.2_@MgO composite spectrum, it is much complex with multiple distinct peaks, including carboxylate stretching at 1362 cm^−1^ and 1584 cm^−1^, which could be an indication of strong interactions between phytochemicals and the MgO surface. The broad O–H stretching peak at 3222 cm^−1^ indicates hydrogen bonding due to the surface hydroxyl group.^24^ In Bi_0.5_@MgO, composite peaks appear at the same positions with slight shifting in carboxylate vibrations, showing a strong effect of Bi^0^ concentration on the bonding environment. On the other hand, in Bi_0.7_@MgO composite, three peaks 866, 1418, and 2960 cm^−1^ show a more uniform structure, so its simplified vibrational mode of material, due to the larger atomic size of Bi^0^, leads to disrupting the MgO structure and homogenizing surface interaction.^25^ The comparison shows a uniform, simplified chemical structure in the Bi_*x*_@MgO composite as the Bi^0^ concentration increases.

**Fig. 1:**
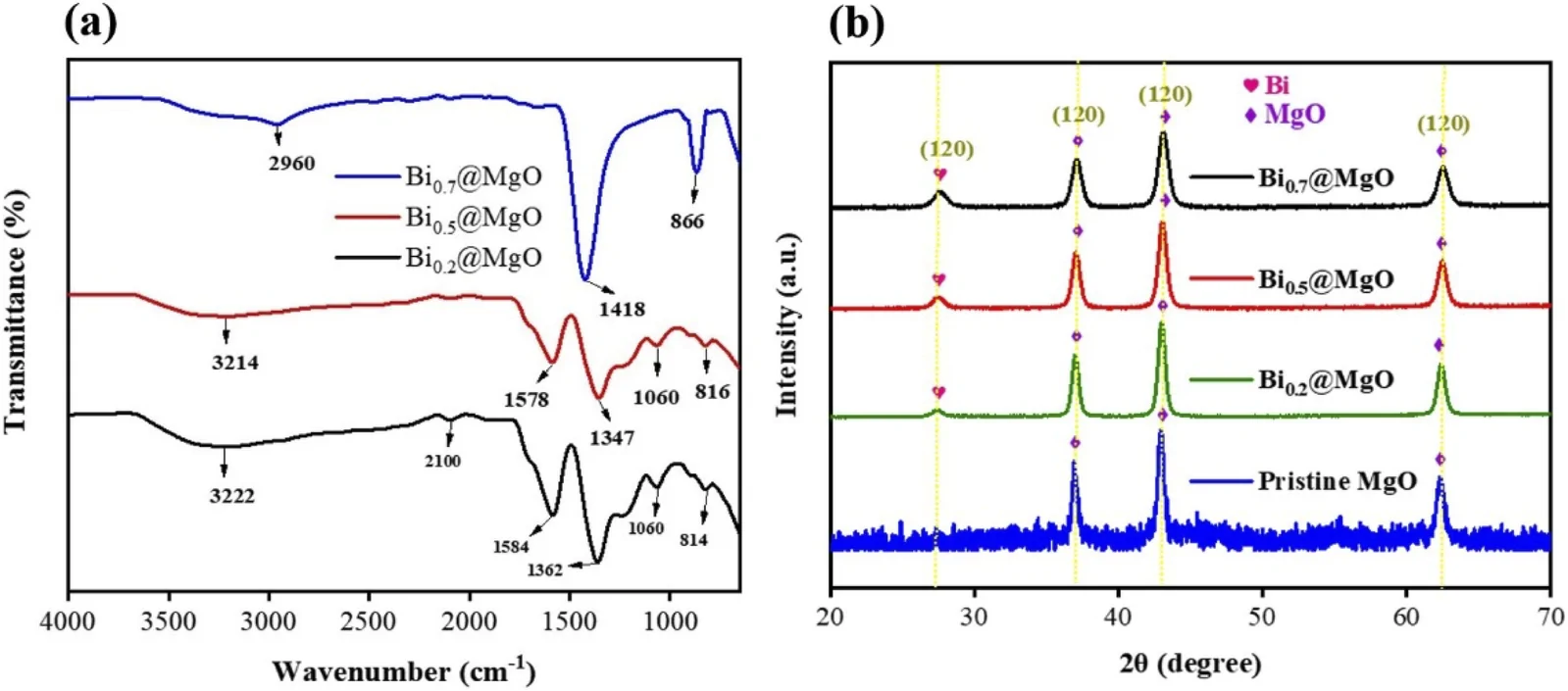
(a) FTIR and (b) XRD pattern of Bi_*x*_@MgO (Bi_0.2_@MgO, Bi_0.5_@MgO, Bi_0.7_@MgO) NPs.

### 3.2 X-ray diffraction (XRD) analysis

X-ray diffraction (XRD) was used to evaluate the crystal structure and purity of pristine MgO and Bi_*x*_@MgO samples, shown in Fig. 1b. The XRD patterns for pristine MgO revealed three major peaks corresponding to the (111), (200), and (220) planes of the cubic periclase MgO structure (JCPDS No. 45-0946). Therefore, high-quality phase-pure cubic MgO has been obtained. After loading with Bi, the crystal lattice of MgO remained the same, as indicated by the continuous presence of all of the characteristic diffraction peaks associated with cubic MgO, while slight peak broadening suggests minor local structural distortion. This demonstrates that mechanochemical modification preserves the crystal structure of MgO. The weak diffraction peak observed at 2*θ* = 27.5° indicates the presence of Bi species^26^ in Bi_*x*_@MgO structure (JCPDS No. 44-246).^27^ However, no intense diffraction peaks corresponding to crystalline Bi_2_O_3_ are detected, indicating that Bi does not form a bulk oxide phase. The preserved crystalline framework together with highly dispersed Bi species provides a structurally stable platform for subsequent electronic structure modulation, as further confirmed by XPS, DRS and photoelectrochemical analyses.

### 3.3 Surface morphology and elemental composition

The surface morphologies of Bi_0.2_@MgO, Bi_0.5_@MgO, and Bi_0.7_@MgO composites synthesized through phytomechanochemical techniques are presented in Fig. 2a–c. The patterns of all samples show irregularly shaped, agglomerated particles with rough surfaces which is the result of high-energy ball milling techniques. With repetitive mechanical collisions, particles are fractured with the surfaces filled with defects and many exposed active sites. Because of the low content of Bi (0.2–0.7 wt%), the SEM images are not able to show clearly defined Bi^0^ NPs which means formation of any clumps of Bi^0^ is ruled out during the solvent-free phytomechanochemical synthesis.

**Fig. 2:**
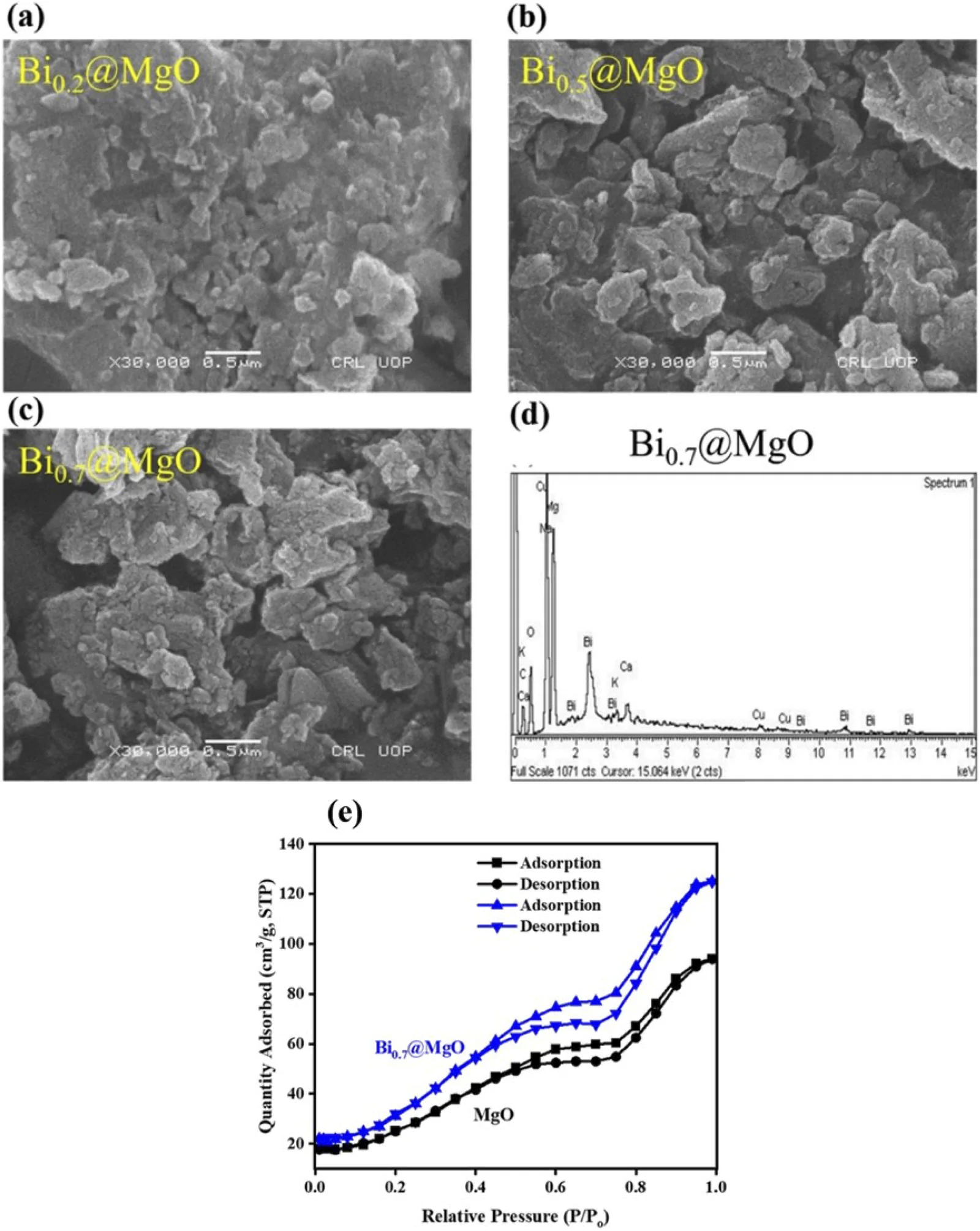
SEM images of (a) Bi_0.2_@MgO, (b) Bi_0.5_@MgO, and (c) Bi_0.7_@MgO, (d) EDX spectrum of Bi_0.7_@MgO, and (e) N_2_ adsorption–desorption isotherms of pristine MgO and Bi_0.7_@MgO.

The EDX spectrum (Fig. 2d) of the Bi_0.7_@MgO shows its components, Mg, O, and Bi, and confirms the successful inclusion of Bi^0^ into the MgO matrix. The weak signals coming from Bi are explained by its low loading, and the strong peak signals correspond to the MgO support. The presence of C, Na, K, Ca, and Cu signals can be explained by the carbon tape, a natural mineral residue from the clove extracts, and the specimen holder used for the SEM.

### 3.4 Surface textural analysis

The investigations of the textural characteristics of pristine MgO and Bi_0.7_@MgO were carried out by N_2_ adsorption–desorption tests (Fig. 2e). In the tests of adsorption over a wide range of relative pressures, it was observed that Bi_0.7_@MgO (surface area: 134.2 m^2^ g^−1^; pore volume: 0.193 cm^3^ g^−1^; average pore diameter: 5.76 nm) demonstrates higher nitrogen sorption compared to pristine MgO (surface area: 104.2 m^2^ g^−1^; pore volume: 0.146 cm^3^ g^−1^; average pore diameter: 5.59 nm) (see SI for calculations). The adsorption–desorption curves also indicate hysteresis and pressure-dependent results which point to the involvement of filling of cavities and mesopore properties in the above measurements. The increased surface area and pore volume suggest enhanced surface accessibility and a greater availability of potential defect sites, providing an indirect indication of oxygen-vacancy-related surface characteristics. Although BET cannot directly confirm oxygen vacancies, their presence was further investigated using XPS and EPR analyses.

### 3.5 Surface chemistry (XPS) and EPR study

Surface chemical states of Bi_0.7_@MgO photocatalyst were investigated using X-ray photoelectron spectroscopy (XPS), as seen in Fig. 3. The high resolution Bi 4f spectrum (Fig. 3a) showed the presence of spin–orbit doublets (4f_7/2_ 156.7 eV and 4f_5/2_ 162.07 eV) unique to metallic Bi^0^,^28^ confirming the successful deposition of Bi onto MgO substrate. The Mg 2p spectrum (Fig. 3b) contained only one peak (49.9 eV) of Mg^2+^ ion,^29^ showing that the chemical state of MgO remained unchanged upon loading its surface with Bi; this is corroborated by XRD results. The deconvoluted O 1s spectrum (Fig. 3c) shows three peaks, namely: lattice oxygen (O_L_ 530.1 eV), oxygen-deficient or defect-associated surface environment (O_V_ 531.6 eV), and surface-adsorbed oxygen (O_C_ 533.7 eV).^30^ The O_V_ peak is the result of the synergetic influence of mechanochemical generation of defects and strong interfacial electronic interactions between metallic Bi and MgO.

**Fig. 3:**
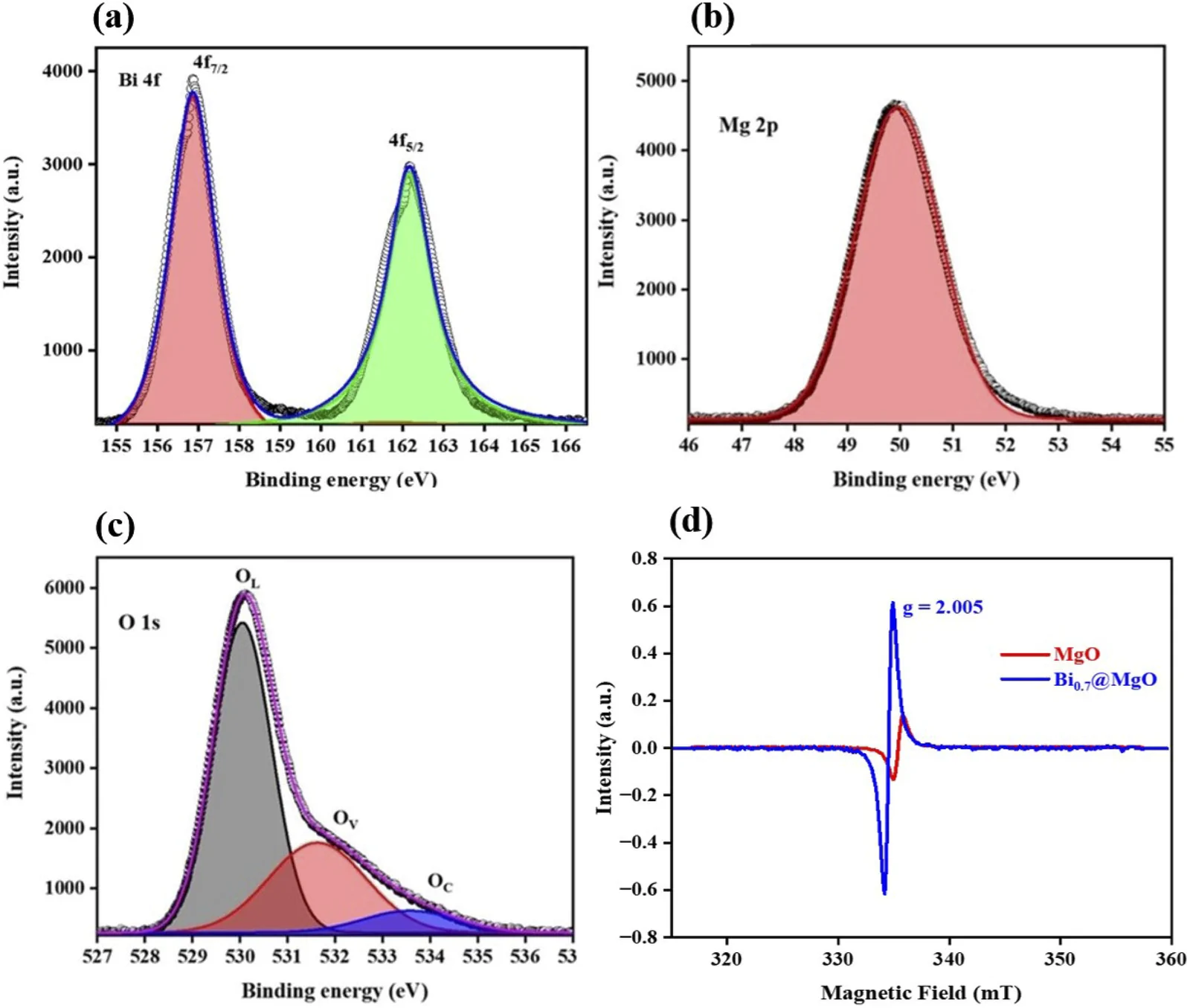
High-resolution X-ray photoelectron spectroscopy (XPS) spectra of the Bi_0.7_@MgO photocatalyst: (a) Bi 4f, (b) Mg 2p, (c) O 1s spectrum, and (d) EPR spectra of pristine MgO and Bi_0.7_@MgO.

In order to investigate defect-related centers, the EPR/ESR activity of Bi_0.7_@MgO was studied (Fig. 3d). The composite gives a significant resonance signal at about (*g* = 2.005), consistent with the presence of paramagnetic centers of defects that may correspond to the vacancies due to oxygen. Nevertheless, the above value does not uniquely support the idea that the observed phenomenon is due to oxygen vacancies and must be studied in relation to O 1s data from XPS. The above findings support the presence of the defect regions in Bi_0.7_@MgO, though it is still impossible to define the specific defect and its concentration based on these characteristics.

It has also been observed that the presence of the oxygen-deficient environment leads to shallow defect states, which enhance the ability to absorb visible light and correspond to the decrease of the optical band gap,^31^ which has also been indicated by DRS analysis. Furthermore, the oxygen-vacancy defects also enable electron trapping and thus lead to less recombination of the electrons and holes,^32^ which can be supported by the drastic decline of PL intensity, as well as to better transfer of charge, provided by lower resistance in the charge transfer, as proved by EIS Nyquist plot and transient photocurrent response. Thus, the interaction of metallic Bi and oxygen-vacancy defects provides better separation and migration of charge carriers, and therefore, better photocatalytic properties concerning hydrogen evolution and antibiotic degradation displayed by Bi_0.7_@MgO.

### 3.6 Optical properties (DRS + Tauc)

The fundamental MgO structure shows strong UV absorption consistent with a wide bandgap semiconductor (Fig. 4a). When Bi is introduced in the MgO matrix, the cutoff of the absorption edge is progressively shifted towards longer wavelengths indicating that visible light absorption has increased.^13^ This redshift is attributed to Bi-induced electronic states and defect levels including those associated with vacancies (specifically O vacancies), both of which alter the electronic structure of MgO resulting in the lower energy of photons being required for excitation and subsequently absorption.^13^

**Fig. 4:**
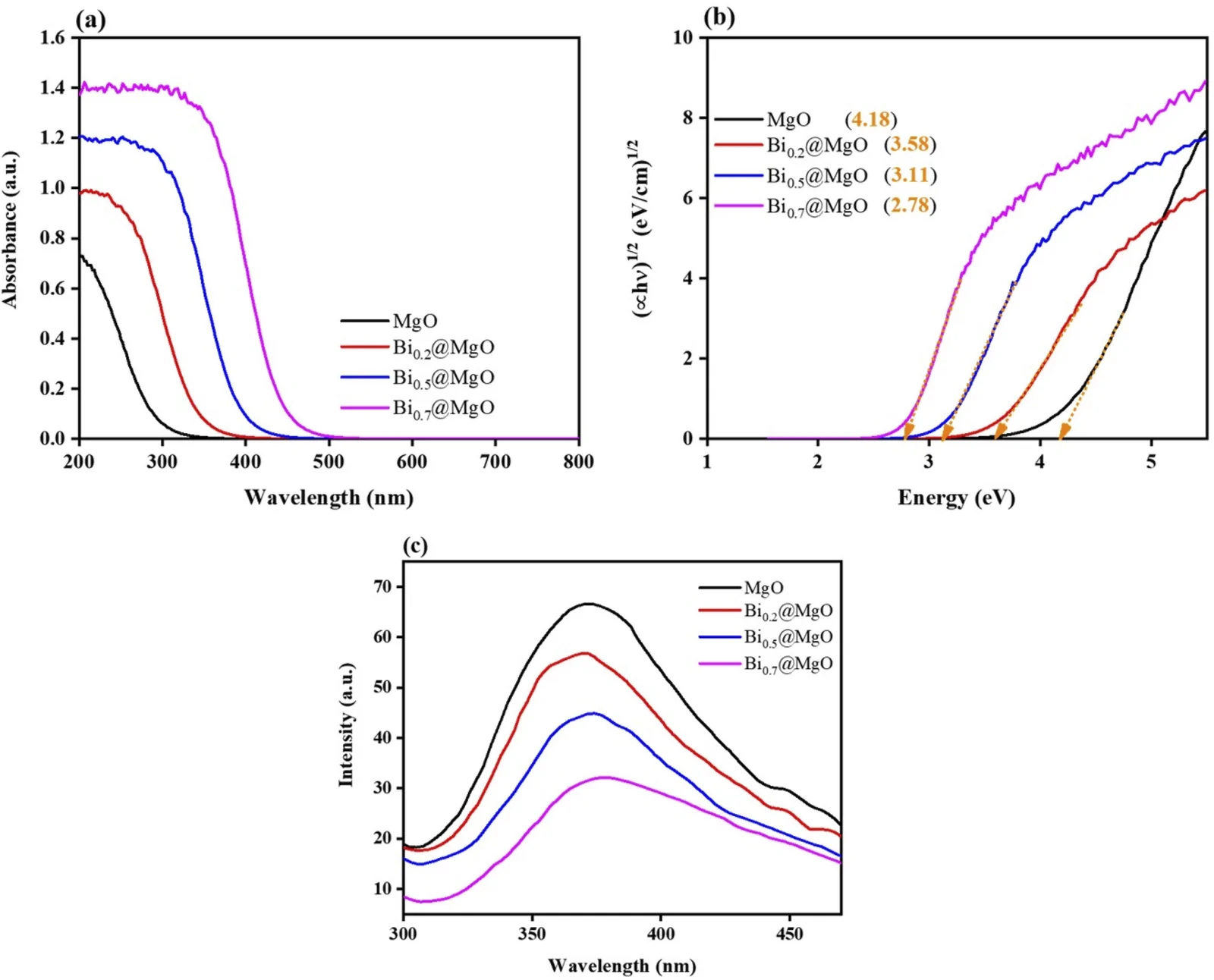
(a) UV-visible diffuse reflectance spectrum of pristine MgO and Bi_*x*_@MgO (*x* = 0.2, 0.5, 0.7) photocatalysts, (b) corresponding Tauc plots, and (c) corresponding photoluminescence emission spectrum.

The optical band gaps determined from Tauc Plots (Fig. 4b) show that the band gap of pristine MgO (4.18 eV) decreased with increasing Bi loading (Bi_0.2_@MgO – 3.58 eV, Bi_0.5_@MgO – 3.11 eV and Bi_0.7_@MgO – 2.78 eV). The successive narrowing of the optical band gaps is an indication that Bi deposition has effectively altered the electronic structure of the host MgO.^33^ The localized energy states are created within the bandgap, thereby facilitating photoreduction of reactants in the visible region of the electromagnetic spectrum. The Bi_0.7_@MgO sample has the smallest optical bandgap and thus potential for enhancing utilization of simulated solar energy producing a positive correlation with expected improvements in photocatalytic efficiency.

### 3.7 Photoelectrochemical properties and charge carrier dynamics

The PL spectrum indicates that there is a broad emission peak from approximately 370 nm to 380 nm in the samples being studied (Fig. 4c). This emission will occur as a result of the recombination of radiatively-recombined, photogenerated charge-carrier pairs.^34^ The intensity of emission decreases from MgO (highest emission) to Bi_0.2_@MgO, then to Bi_0.5_@MgO, and finally to the lowest intensity, Bi_0.7_@MgO. The decrease in intensity exhibited by the Bi-loaded samples compared to the pristine MgO indicates that Bi reduces the recombination of generated charge carriers. Bi_0.7_@MgO exhibits the lowest PL intensity and the highest charge separation efficiency as compared to Bi_0.2_@MgO and Bi_0.5_@MgO. Based on Nyquist plots (Fig. 5a), Bi_0.7_@MgO exhibited much smaller semicircles than pristine MgO (which suggests that *R*_ct_ has greatly diminished); thus, there is evidence for interfacial resistance reductions due to electronic conduction. The increased charge/electron transport properties are likely due to both the increase in charge carrier mobility caused by Bi induced electronic states, as well as the reduction in electron–hole recombination rates,^35^ because of the increased charge utilization during photocatalytic production of hydrogen and degradation of antibiotics by using Bi_0.7_@MgO. From the positive slopes of Mott–Schottky plots (Fig. 5b), it is confirmed that MgO and Bi_0.7_@MgO have n-type semiconducting properties, with flat-band potentials of −0.45 V and −0.35 V *vs.* Ag/AgCl, respectively. This will be equal to −0.25 V and −0.15 V *vs.* NHE (Fb +0.197 V *vs.* NHE). Given that the conduction band edge of an n-type semiconductor is expected to be more negative (−0.20 V) than the flat-band potential,^36^ this means that Bi_0.7_@MgO has a conduction band (−0.15 V *vs.* NHE) which is more negative than the H^+^/H_2_ redox potential. Therefore, the favorable band alignment suggests that Bi_0.7_@MgO will not only photocatalytically generate H_2_ but also produce enough reactive oxygen species to degrade antibiotics. Photocurrent density generated by Bi_0.7_@MgO was enhanced compared to MgO as evidenced by LSV curves (Fig. 5c), revealing significantly greater photocurrent density under illumination. In the dark, there is very little or no current produced. The pristine MgO and Bi_0.7_@MgO exhibited extremely rapid and repeatable on/off photocurrent cycles (Fig. 5d). Bi_0.7_@MgO photocatalyst exhibited approximately four to five times higher photocurrent intensity levels compared to pristine MgO due to effective charge carrier separation as well as transport produced during light illumination (photogenerated charge carriers) and the long-term preservation of those produced (photogenerated) charges, where the stable and reproducible performance of the photocatalysts in terms of their response was maintained without significant degradation over time.

**Fig. 5:**
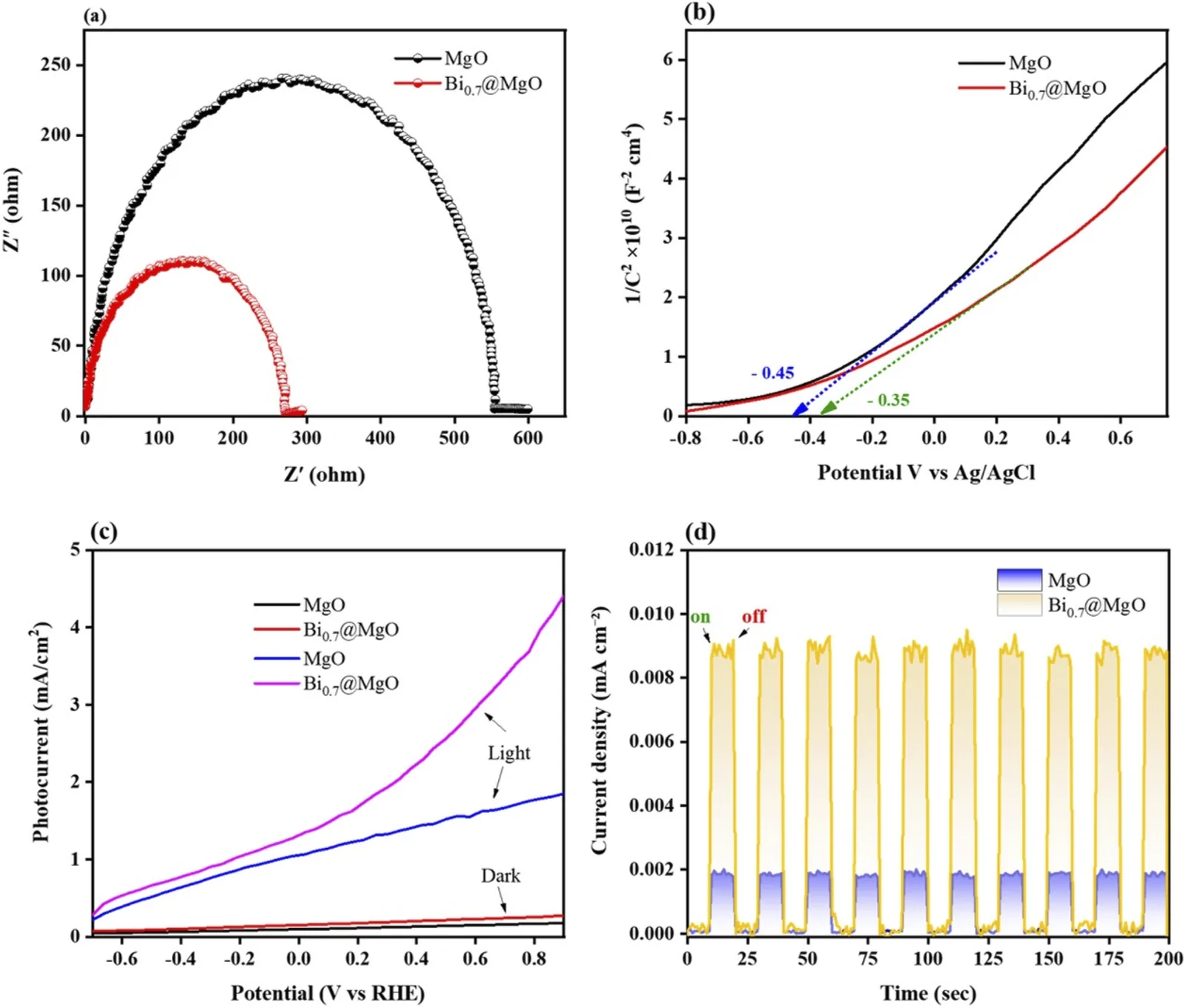
(a) Electrochemical impedance spectroscopy (EIS) Nyquist plots, (b) Mott–Schottky plots, (c) linear sweep voltammetry (LSV) curves under dark and simulated solar-light irradiation, and (d) transient photocurrent response under intermittent light illumination.

### 3.8 Photocatalytic hydrogen evolution activity of pristine MgO

Fig. 6a and b represent the cumulative and per-hour H_2_ activity of MgO. As shown in Fig. 6a, the cumulative amount of hydrogen produced after six hours is 67.19 mmol g^−1^. This suggests that the rate of production of photogenerated charge carriers is little bit sufficient to reduce protons to molecular H_2_. Furthermore, the hourly evolution of H_2_, shown in Fig. 6b, appeared to increase during the first four hours of irradiation before reaching a plateau (17.02 mmol g^−1^ h^−1^). After this, there are expected to be some factors causing the H_2_ evolution rate to decrease (11.19 mmol g^−1^ h^−1^). This could be due to the accumulation of reaction intermediates on the catalyst surface that block catalytic sites; the presence of intermediates may also increase the likelihood of electron–hole recombination. The disparity (especially after 4 hours) between the H_2_ production capacity of pure MgO and Bi_*x*_@MgO may arise from the discrepancy in their electronic properties. It is suggested that the drop in the H_2_ production rate of pure MgO around 4 hours may be due to the partial occupation of active sites and enhanced electron–hole recombination. Alternatively, the superior light absorption and charge transfer property of Bi_*x*_@MgO, as demonstrated by DRS, PL, EIS, and the obtained results of transient photocurrent, may lead to better photocatalytic performance. Nevertheless, this does not serve as the only basis for affirming that Bi modifications prevent deactivation of the surface in prolonged irradiation. Based on the H_2_ production rate and the area of the reactor directly exposed to lamp (approximately 0.001 m^2^), the AQY is given in Table 1.

**Fig. 6:**
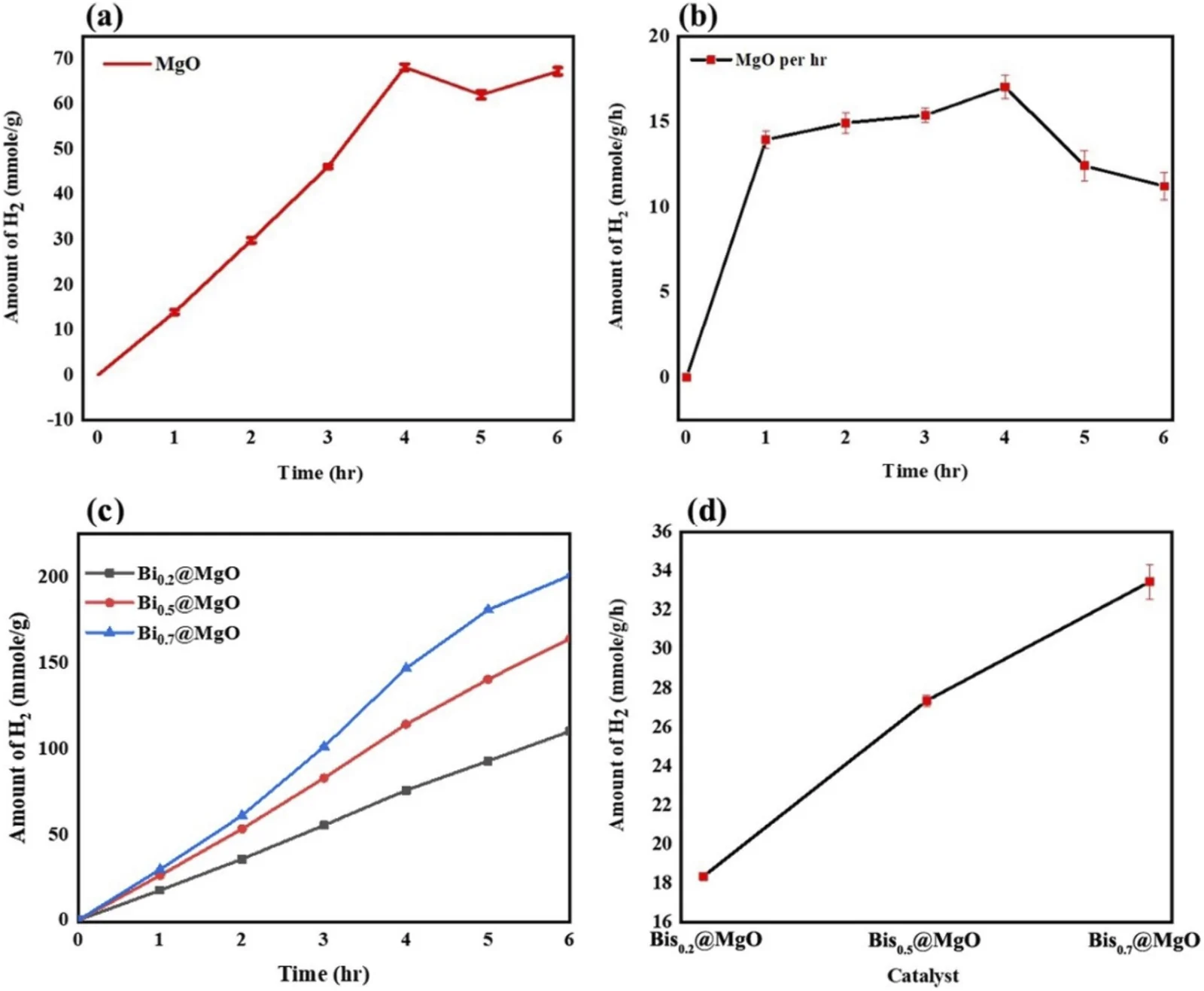
(a) Time-dependent cumulative H_2_ production over pristine MgO, (b) hourly H_2_ evolution rate of pristine MgO, (c) effect of Bi loading (Bi_0.2_@MgO, Bi_0.5_@MgO, and Bi_0.7_@MgO) on the hydrogen evolution rate. (d) Optimization of catalyst loading for photocatalytic H_2_ generation. Error bars denote the standard deviation from triplicate measurements.

**Table 1:** Photocatalytic hydrogen evolution activity and AQY of MgO and Bi_*x*_@MgO photocatalysts

Photocatalyst	H_2_ evolution (mmol g^−1^ 6 h^−1^)	Total H_2_ evolved (mmol 6 h^−1^)	H_2_ evolution rate (mol s^−1^)	AQY (%)
MgO	67.19	0.34	1.56 × 10^−8^	0.88
Bi_0.2_@MgO	110.07	0.55	2.55 × 10^−8^	1.44
Bi_0.5_@MgO	164.06	0.82	3.80 × 10^−8^	2.15
Bi_0.7_@MgO	200.65	1.00	4.64 × 10^−8^	2.63

#### 3.8.1 Optimization of Bi (wt%) loading on MgO

The influence of Bi loading and catalyst loading on the performance of MgO for photocatalytic H_2_ production, is shown in Fig. 6c and d. The presence of Bi increased the amount of hydrogen produced as the concentration of Bi increased. Of the investigated compositions, Bi_0.7_@MgO produced the highest H_2_ rate (200.65 mmol g^−1^) in 6 h. The increase in generated hydrogen was from the modification of the electronic structure of MgO caused by the addition of Bi, instead of from an increased number of active sites. Overall, the following trend has been observed. Bi_0.7_@MgO > Bi_0.5_@MgO > Bi_0.2_@MgO (Fig. 6d). However, since compositions with a Bi content higher than 0.7 wt% have not been tested, it cannot be ruled out that better results can be obtained with higher Bi concentration. Although increasing Bi content can bring extra active sites and thus improve the properties of the compound, it is likely that too much Bi may prevent the appearance of active sites or lead to charge carrier recombination. Therefore, it can be stated that the identified Bi level of 0.7 wt% is optimal among the compositions examined in this study, rather than a universally established optimum.

#### 3.8.2 Effect of Bi_0.7_@MgO concentration

Fig. 7a and b displayed the effect of the most efficient catalyst (Bi_0.7_@MgO) loading on the rate of hydrogen evolution. It is clearly observed that the catalyst's dose influences photocatalytic performance by affecting the number of photons used and the size of its active surface area. The amount of hydrogen produced from the photocatalytic reaction will increase with the catalyst loading up to 5 mg (200.65 mmol g^−1^), since there will be more catalytic sites available for light to be absorbed and for surface redox reactions to occur. Increasing the catalyst loading above 5 mg will decrease hydrogen evolution (90.72 mmol g^−1^ using 7 mg of catalyst) because, with the increase in suspension turbidity, light will be scattered, and because of shielding effects, photons will be unable to effectively penetrate into the suspension where electrons can be charged – thus preventing efficient charge transport. Therefore, 5 mg makes up the optimal dose of catalyst to balance light absorption, surface reaction kinetics, and charge transfer and allow Bi_0.7_@MgO to achieve maximum hydrogen evolution efficiency.

**Fig. 7:**
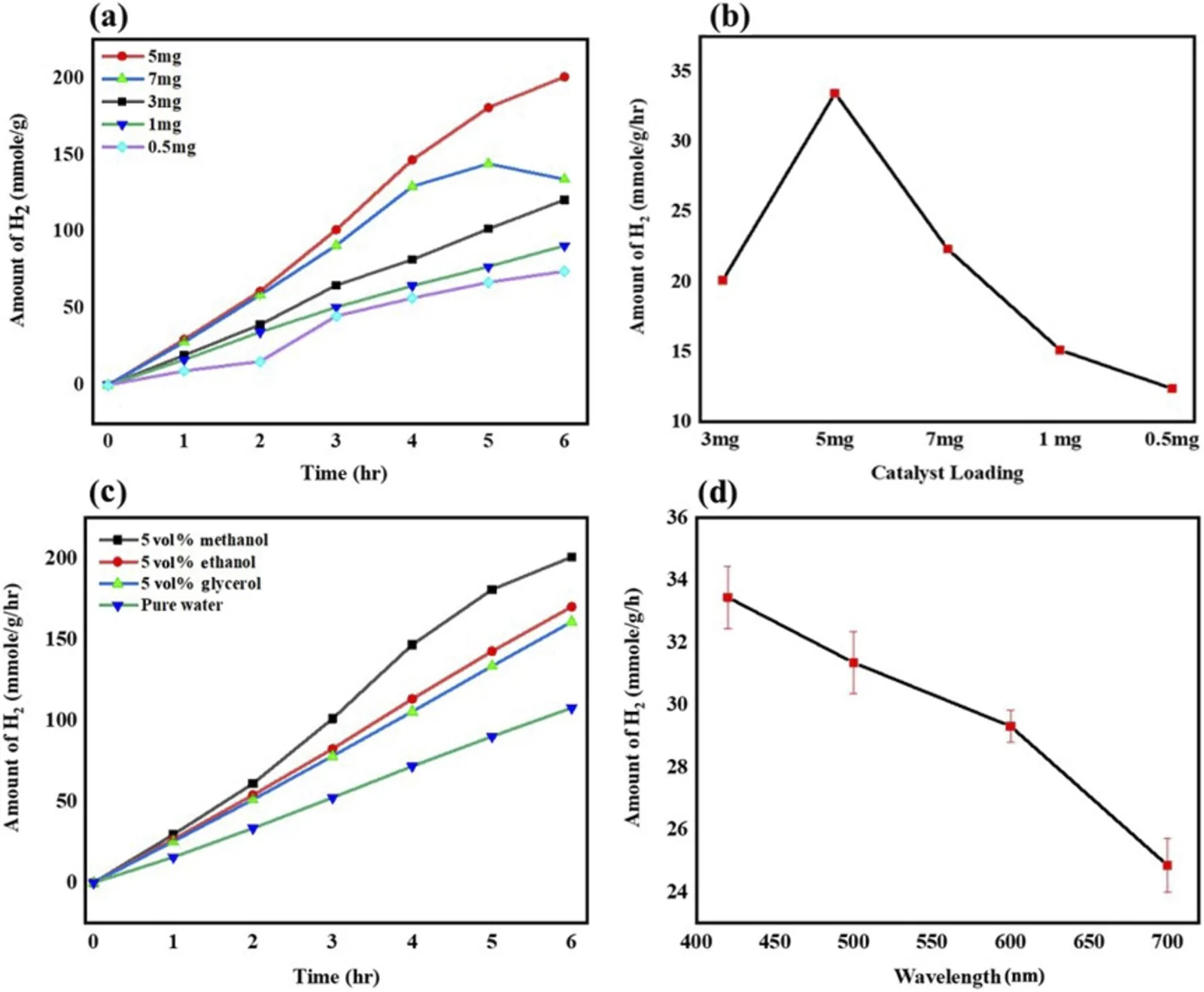
(a) Optimization of catalyst dosage (0.5–7 mg), (b) per hour calculations. (c) Effect of different sacrificial agents (5 vol% methanol, ethanol, glycerol, and pure water) (d) effect of irradiation wavelength on the optimized Bi_0.7_@MgO photocatalyst. Data is presented as mean ± standard deviation from triplicate measurements.

#### 3.8.3 Effect of sacrificial agent

Fig. 7c compares the photocatalytic hydrogen evolution performance obtained using different sacrificial reagents. The hydrogen evolution activity follows the sequence:Methanol > ethanol > glycerol > pure water

Methanol performs well as a sacrificial agent due to its low activation energy and ability to consume electrons/hole generated through photochemical oxidation reaction. As a result of rapidly oxidizing methanol, the number of holes and electrons is no longer able to combine but rather remains separated and able to increase the time duration for conduction band electrons to reduce protons *via* photocatalysis. Therefore, by adding methanol, the maximum amount of hydrogen is produced. Pure water does not act as an efficient hole scavenging agent, even though Bi_0.7_@MgO has superior electronic properties. These results provide evidence that, in addition to the use of band gap modified components, efficient photogenerated hole consumption is also an equally important factor in maximizing photocatalytic H_2_ production.

#### 3.8.4 Effect of irradiation wavelength

A 300 W Xe lamp equipped with interchangeable band-pass filters (420, 500, 600, and 700 nm) was employed to investigate the wavelength-dependent activity. Fig. 7d shows the wavelength dependence of hydrogen evolution performance for Bi_0.7_@MgO. The amount of hydrogen produced by Bi_0.7_@MgO increases maximally, *i.e.*, exhibits the highest performance (33.44 mmol g^−1^ h^−1^) when exposed to light with a wavelength of 420 nm. The amount of H_2_ produced thus decreases as the irradiation wavelength is increased towards 700 nm. The decreasing energy of photos upon increased wavelength (*i.e.*, to produce the charge carriers) generates fewer charge carriers under longer wavelengths, ultimately decreasing the amount of hydrogen produced by Bi_0.7_@MgO due to the lack of sufficient energy to produce sufficient charge carriers.

#### 3.8.5 Impact of temperature

The influence of reaction temperature on photocatalytic hydrogen evolution was investigated over the temperature range of 25–45 °C while maintaining all other reaction parameters constant. As shown in Fig. 8a, the H_2_ evolution rate exhibits a volcano-type dependence on reaction temperature. At 25 °C, the photocatalytic activity is relatively low (29.60 mmol g^−1^ h^−1^), indicating that the surface redox reactions are kinetically limited under ambient conditions. The highest rate of production of hydrogen (33.40 mmol g^−1^ h^−1^) occurs at 35 °C, indicating that this temperature produces the best balance between separating the electronic charges created through light energy and how fast the electronic charges can react with hydrogen ions. Once the temperature exceeds 40 °C to 45 °C, there is a slow decreasing rate of H_2_ production due to thermal charge carrier recombination, decreased adsorptive capacity of the catalyst surfaces, as well as lower solubility of the dissolved gases at elevated temperature, all of which limit photocatalysis efficiency.

**Fig. 8:**
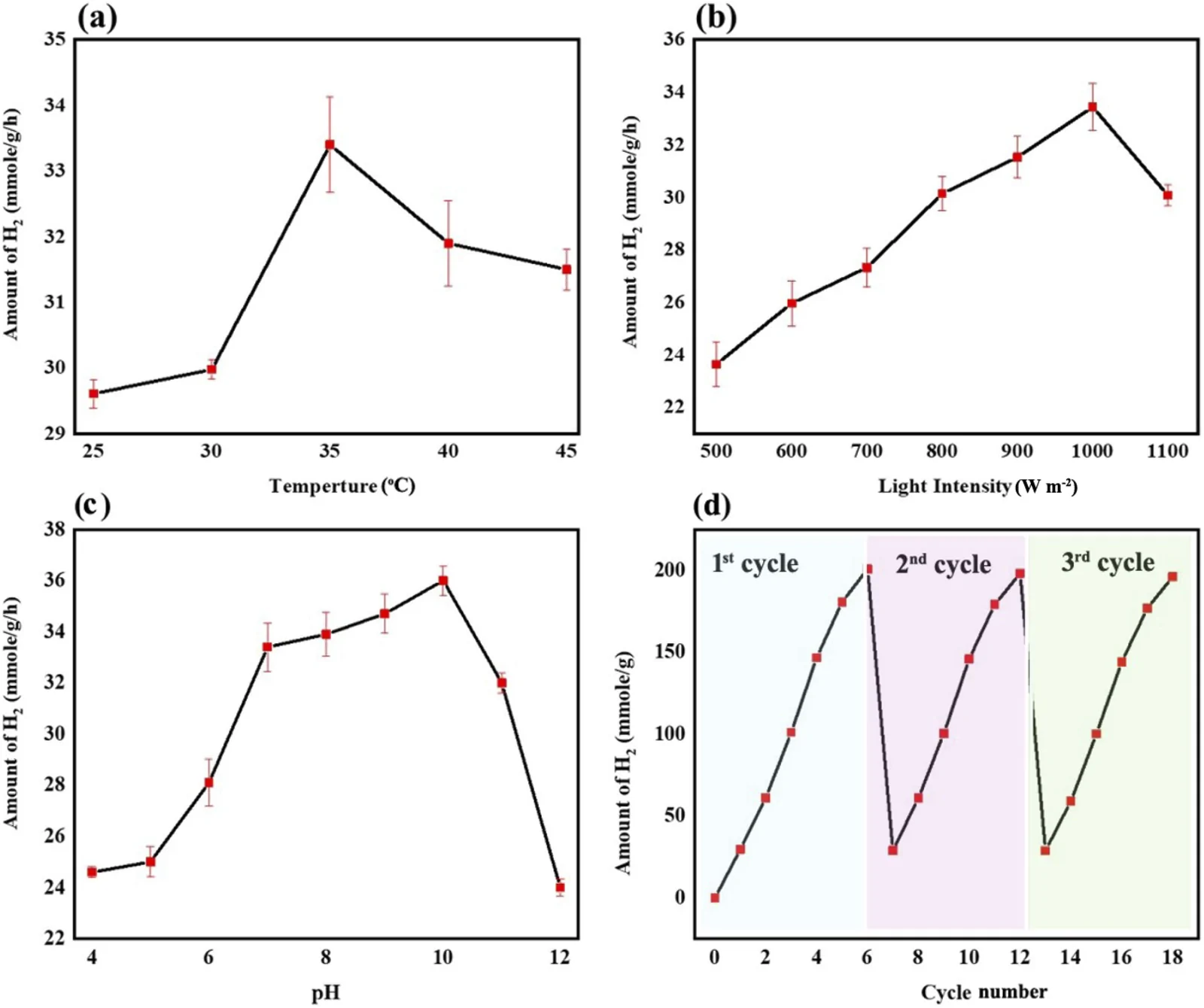
(a) Effect of reaction temperature, (b) influence of incident light intensity, (c) effect of pH, (d) recyclability analysis of Bi_0.7_@MgO. Error bars represent the mean ± standard deviation of three independent experiments.

#### 3.8.6 Impact of light intensity

When the light intensity is low (500), H_2_ is generated in limited amounts (23.64 mmol g^−1^ h^−1^) due to only small portions of the catalyst active sites being exposed to create an electron–hole pair (Fig. 8b). As a result, the number of electrons generated by photons that are available for the reduction of protons is very low. As the light intensity is increased from 600 to 1000 the amount of H_2_ produced continues to increase in a linear fashion due primarily to the increased number of charge carriers produced. This allows for a higher probability of interfacial reduction occurring before the excited electrons recombine. This results in the highest amount of hydrogen production (33.4 mmol g^−1^ h^−1^) at 1000 W m^−2^ light intensity. An increase from 1000 W m^−2^ to 1100 W m^−2^ causes larger reductions in photocatalytic activity due to rapid production of electron–holes. This results in a significant increase in the bulk and surface recombination of electron–holes. Another significant factor involved is high intensity light resulting in localized photothermal effects that can create unstable adsorption equilibrium and therefore reduce the quantum efficiency of charge separation.

#### 3.8.7 Impact of pH

Fig. 8c represents the dependance of H_2_ production on pH of the reaction medium and kinetics at the interface of catalyst/reactant change as the acidity of the solution changes (pH 4–12). Hydrogen production was slow (24.60–28.10 mmol g^−1^ h^−1^) in acidic conditions (pH 4–6) because even though there is a lot of H^+^ to react with the positively charged catalyst/film surface repelled the sacrificial species from attaching to its surface and therefore limited the available H^+^ and slowed the kinetics of H_2_ generation. As the pH of the solution increased from 6–10, H_2_ production steadily increased and peaked (28.10–36.00 mmol g^−1^ h^−1^) at pH 10. At pH = 10, Bi_0.7_@MgO has favorable surface properties that help reactants adsorb efficiently and allow electron flow from the photo-excited electron to occur rapidly at the site of reduction (H^+^) for producing H_2_. Additionally, the alkaline medium limits the extent of photocorrosion and therefore extends the lifetime of the photo-excited electrons, which will enhance photocatalytic activity/efficiency. The production of H_2_ shows a significant decline (24 mmol g^−1^ h^−1^) at pH 12. The excessively alkaline conditions change the surface chemistry of the catalyst by decreasing the number of reducible protons available for producing H_2_ and by creating competition from hydroxide ions to adsorb on the same sites as protons, thus partially blocking the active sites for H_2_ production.

#### 3.8.8 Reusability and stability

The Bi_0.7_@MgO photocatalyst was tested for its long-term stability and recyclability based on three consecutive photocatalytic H_2_ evolution cycles under identical reaction conditions. After each cycle of photocatalytic H_2_ evolution, the photocatalyst was recovered by centrifugation, then thoroughly washed with distilled water and ethanol, dried, and reused without any other treatment. As shown in Fig. 8d, the photocatalyst maintained its high photocatalytic activity through all three cycles of the photocatalytic H_2_ evolution tests. The first cycle produced approximately 200.65 mmol g^−1^ of H_2_ after 6 h of illumination, while the second and third cycles produced about 197.93 mmol g^−1^ and 195.87 mmol g^−1^, respectively. The minimal decrease in H_2_ production (<6%) over multiple uses indicates the structural integrity and chemical stability of the Bi_0.7_@MgO photocatalyst is very good. The slight reduction in H_2_ production is likely due, in part, to unavoidable catalyst losses during the recovery process and to partial surface coverage with reaction intermediates, rather than degradation of the catalyst materials. The very good cycling performance of this photocatalyst suggests that the loading of Bi effectively stabilized the MgO framework, maintained active surface sites, and allowed for efficient charge separation during long periods of photocatalytic operation. These findings confirm the very good durability of the Bi_0.7_@MgO photocatalyst for H_2_ production.

To further investigate the stability of Bi_0.7_@MgO photocatalyst structure during multiple uses, post-recycling XRD analysis has been carried out on the retrieved catalyst. The results (Fig. S1) indicate that Bi_0.7_@MgO, after completion of the recycling process, continues to exhibit characteristic diffraction peaks at about 2*θ* = 27.5°, 37°, 43°, and 62.5° ascribed to (120), (111), (200), and (220) plane positions, respectively. These diffraction peaks are in line with the results of fresh catalysts, suggesting that the main crystalline phases in the recycled sample underwent little changes in terms of structure after the photocatalytic recycling process. The preservation of the various characteristic peaks confirms that the structure of Bi_0.7_@MgO is stable enough and is in accordance with the good recyclability of this photocatalyst.

### 3.9 Effect of catalyst loading on photocatalytic degradation of antibiotics

The potential of the highly efficient photocatalyst Bi_0.7_@MgO was further analyzed through the degradation of various potent antibiotics (AMX, CFX, AZM, and CIP) (Fig. 9). As the amount of catalyst increased, the efficiency of the degradation process increased until reaching some maximum point; this indicates that an equilibrium exists between the amount of photons being absorbed, the number of active sites available, and the efficiency of charge transfer. If there is too little catalyst present, there will not be enough active sites available to absorb the available light energy or attract/adhere to the pollutant molecules. Increasing the catalyst quantity will improve the amount of light absorbed and provide additional reaction sites.

**Fig. 9:**
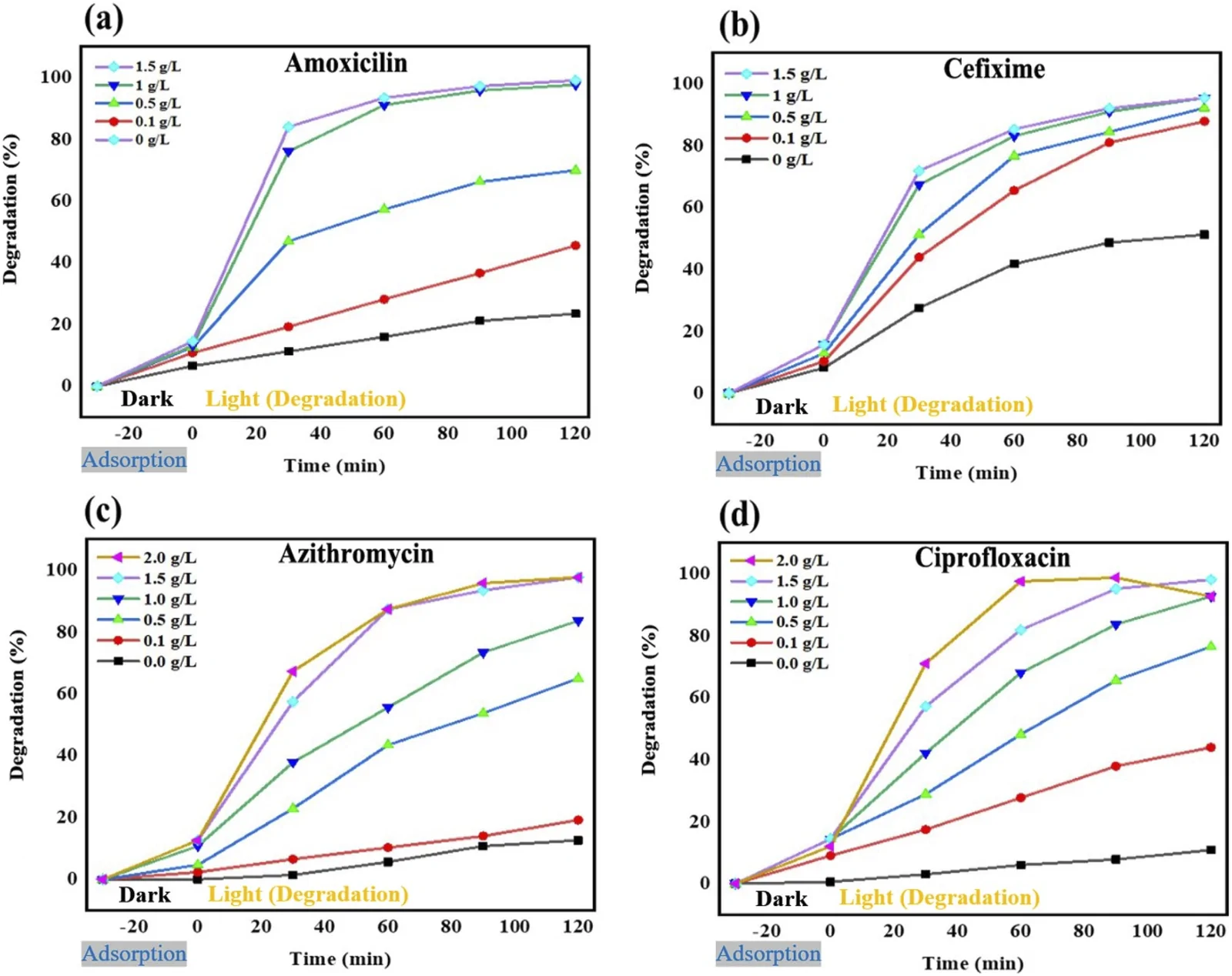
Effect of Bi_0.7_@MgO photocatalyst loading on light-driven degradation of (a) amoxicillin, (b) cefixime, (c) azithromycin, and (d) ciprofloxacin. Degradation efficiency (%) was evaluated at different catalyst loadings as a function of irradiation time under identical experimental conditions.

#### 3.9.1 Effect of initial antibiotic concentration

The performance of the optimized Bi_0.7_@MgO photocatalyst in the photodegradation of (AMX, CFX, AZM, and CIP) was evaluated in the range of 50 to 1000 µg L^−1^ (Fig. 10). It was found that the degradation efficiency for all antibiotics decreased as initial concentration was increased; however, degradation continued through the duration of irradiation. The photocatalyst rapidly degraded the antibiotics at lower concentrations (50 to 100 µg L^−1^) as the Bi_0.7_@MgO photocatalyst absorbed the pollutants from the water and converted light to activate the degradation process.

**Fig. 10:**
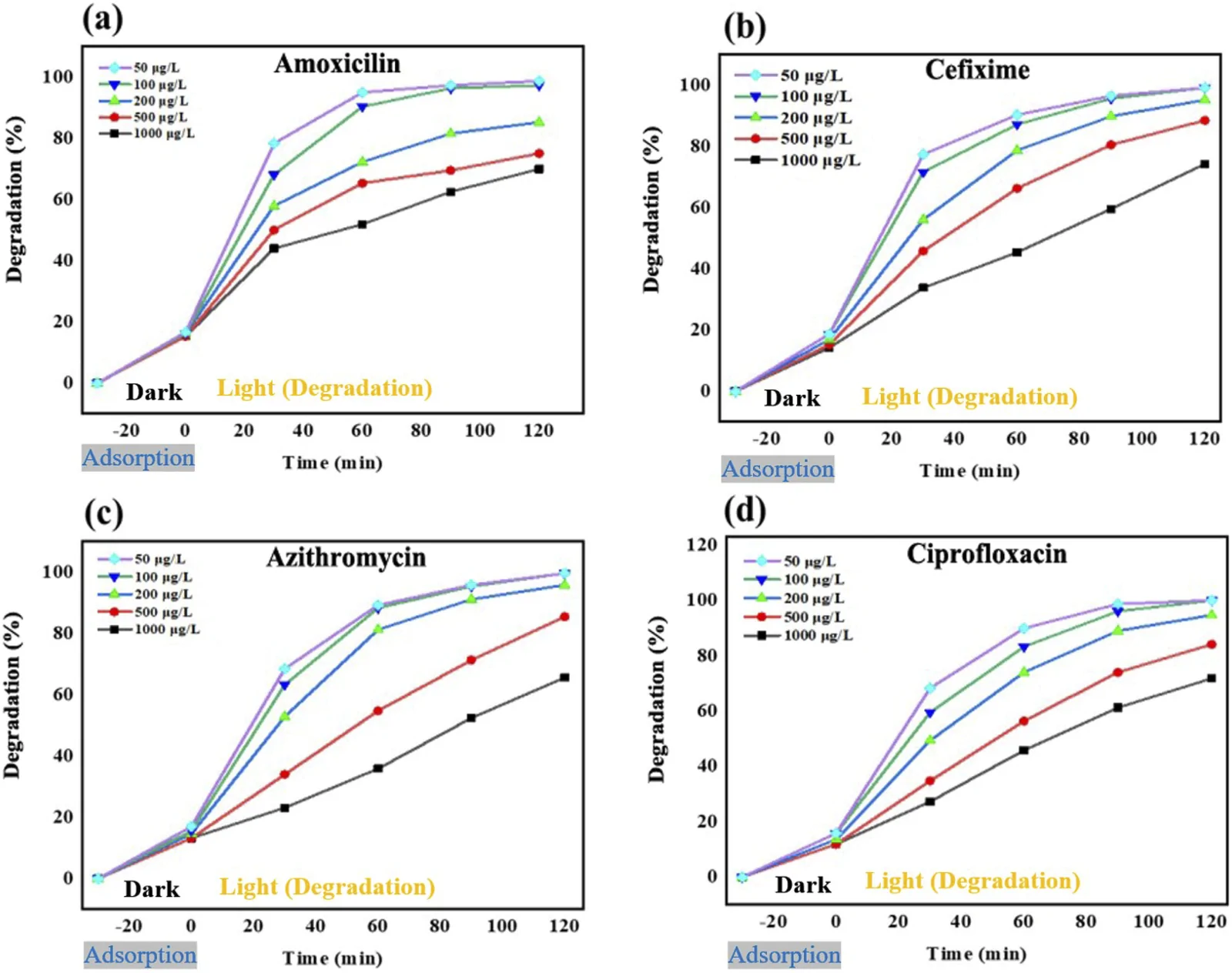
Effect of initial antibiotic concentration on photocatalytic degradation of (a) amoxicillin, (b) cefixime, (c) azithromycin, and (d) ciprofloxacin over the Bi_0.7_@MgO photocatalyst. Degradation efficiency (%) is presented as a function of irradiation time for different initial antibiotic concentrations.

As the initial concentration of the antibiotics increased, there was a decrease in the degradation efficiency. This is likely due to the saturation of the catalytic active sites as well as competition from the degradation intermediates for adsorption on the catalyst. As the concentration increased, there was also less light penetration and therefore less photon generation within the catalyst, causing the degradation efficiency to be related to the limitations of adsorption and the limitations in optical properties, not to a loss of inherent photocatalytic activity of Bi_0.7_@MgO.

The observed concentration dependence is consistent with the Langmuir–Hinshelwood mechanism whereby degradation rates are affected by both surface adsorption and intrinsic photocatalytic activity. At lower concentrations, because of efficient use of active sites, pseudo-first-order reaction kinetics will occur. At higher concentrations, surface saturation will occur, resulting in a reduced reaction rate. All antibiotics displayed the same general trend, but there were differences in their degradation efficiency due to the molecular structures of the antibiotics. Both AMX and CFX showed fast degradation because they have good polar functional groups which permit strong adsorption; CIP also showed good removal due to its amphoteric characteristics. Conversely, AZM showed lower degradation efficiency because of its bulky structure which limits adsorption.

#### 3.9.2 Effect of solution pH on photocatalytic degradation of antibiotics

The effect of pH on the photodegradation efficiency of antibiotics (AMX, CFX, AZM, and CIP) in the presence of the optimized Bi_0.7_@MgO photocatalyst indicate that efficiency was best at mildly acidic pH (5), lower at neutral pH (7), and lowest at alkaline pH (9), as seen in Fig. 11. Increased degradation efficiency due to favorable interactions between Bi_0.7_@MgO and antibiotics at pH 5 improved both the adsorption of antibiotics and charge transfer. Active Bi_0.7_@MgO at neutral pH had lower degradation efficiency than at acidic pH as well as the surface charge of Bi_0.7_@MgO and the speciation of antibiotics changed. The decrease in photocatalytic activity due to electrostatic repulsion against hydroxide ions at pH 9 in comparison with pH 5 enabled the Bi_0.7_@MgO photocatalyst to remain active by harvesting visible light but have detrimental effects on both adsorption and reaction kinetics upon contact with the photocatalyst.

**Fig. 11:**
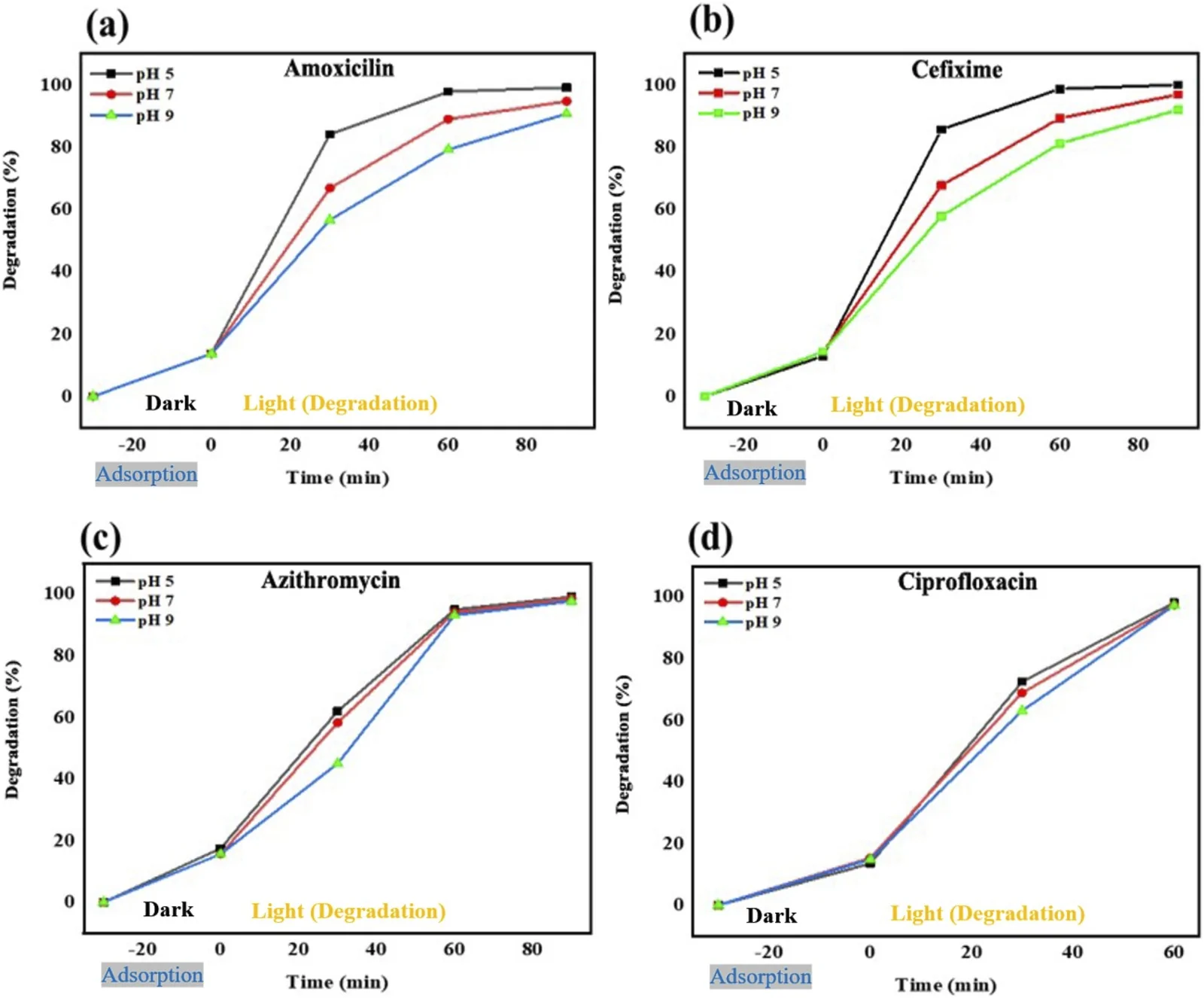
Effect of solution pH on photocatalytic degradation of (a) amoxicillin, (b) cefixime, (c) azithromycin, and (d) ciprofloxacin over the Bi_0.7_@MgO photocatalyst. Degradation efficiency (%) is shown as a function of irradiation time at pH 5, 7, and 9 under identical reaction conditions.

While each of these antibiotics has structural differences between them, they all show pH-dependent activity in pattern. AMX and CFX are more susceptible to fluctuations in pH due to having multiple ionizable groups, whereas CIP breaks down consistently due to its amphoteric behavior. Additionally, AZM was found to be slower to break down owing to steric hindrance. In conclusion, the pH-dependent results indicate that Bi_0.7_@MgO's photocatalytic activity is due to the electronic configuration which enhances light absorption, decreases charge carrier recombination, and enhances electron transport especially under mildly acidic conditions. Therefore, this catalyst has significant potential for environmental remediation and sustainable fuel production.

#### 3.9.3 Photocatalytic stability and reusability

The Bi_0.7_@MgO photocatalyst was tested for its functionality and reusability through six degradation cycles using various antibiotics (AMX, CFX, AZM, and CIP) (Fig. 12). The results showed that it maintained high efficiency with only slight reductions in effectiveness, indicating its potential for long-term use due to its structural and electronic properties. The modifications to MgO's electronic structure enhanced Bi_0.7_@MgO's performance without compromising its crystalline lattice. This confirms its structural integrity and minimal impact from degradation intermediates. This stability suggests that Bi_0.7_@MgO would also be durable for photocatalytic hydrogen evolution. Overall, Bi_0.7_@MgO exhibits exceptional photocatalytic activity, structural integrity, and long-term reliability, facilitating both sustainable hydrogen production and antibiotic degradation.

**Fig. 12:**
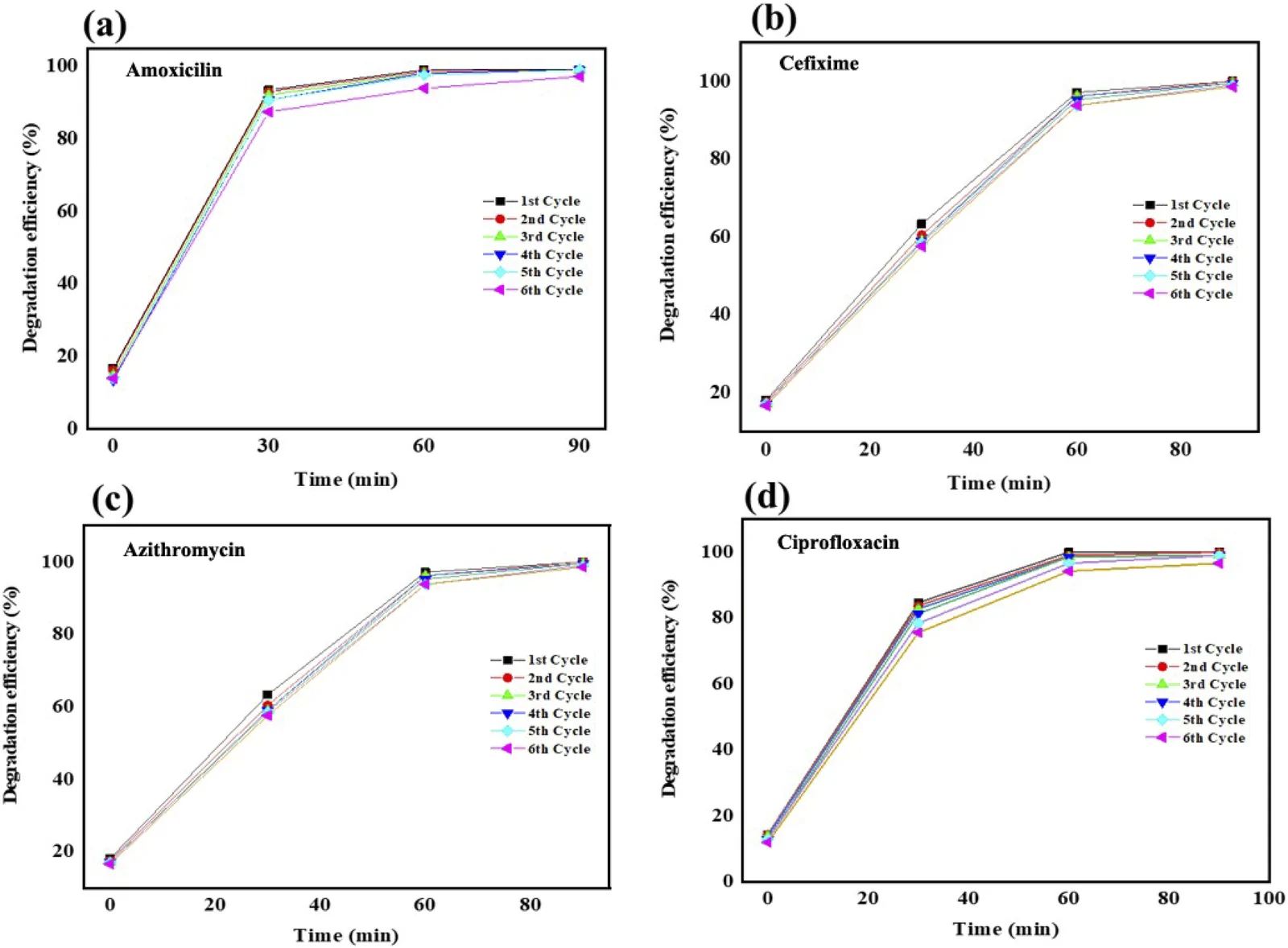
Recycling performance of the Bi_0.7_@MgO photocatalyst for the degradation of (a) amoxicillin, (b) cefixime, (c) azithromycin, and (d) ciprofloxacin over six consecutive photocatalytic cycles.

### 3.10 Correlation between photocatalytic H_2_ evolution and antibiotic degradation

Photocatalytic degradation studies have been conducted to complement studies of H_2_ generation and confirm the intrinsic photocatalytic properties of Bi_0.7_@MgO during oxidative reactions (*e.g.*, photocatalytic degradation of organic materials). Although hydrogen production and photocatalytic degradation of antibiotics involve very different applications. But both processes are based on the same fundamental photophysical events, *i.e.*, (1) light absorption, (2) generation of charge carriers, (3) separation of charge carriers, (4) migration of charge carriers, and (5) interfacial redox reactions (*i.e.*, reduction of the photocatalyst and oxidation of the organic material). As a result, the exceptional performance of Bi_0.7_@MgO in both hydrogen generation and organic degradation is verification that the presence of Bi in the photogenerated semiconductors increases the use of photogenerated electrons and holes by reducing their recombination. The same electronic structure that promotes hydrogen evolution also enhances antibiotic degradation by efficiently separating photogenerated electrons and holes, confirming the multifunctional nature of Bi_0.7_@MgO. Furthermore, the correlation between hydrogen production and antibiotic degradation indicates that the Bi-modulated electronic structure is the main reason for the multifunctional photocatalytic activity of Bi_0.7_@MgO.

### 3.11 Possible hydrogen production photocatalytic mechanism of Bi_*x*_@MgO

The enhanced photocatalytic performance of Bi_0.7_@MgO originates from the synergistic effects of interfacial electronic modulation induced by metallic Bi, oxygen-vacancy defects, and improved charge-carrier separation. As illustrated in Fig. 13, pristine MgO possesses a wide band gap (4.18 eV) with conduction-band (CB) and valence-band (VB) potentials located at −0.25 V and +3.93 V *vs.* NHE, respectively. After Bi loading, the band gap narrows significantly to 2.78 eV, while the CB and VB positions shift to −0.15 V and +2.63 V *vs.* NHE, respectively. Interfacial electronic states associated with Bi modification and oxygen-vacancy defects likely introduce intermediate energy levels within the band gap, facilitating charge transfer. Upon light irradiation, electrons are excited from the VB to the CB, leaving corresponding holes in the VB:Bi_0.7_@MgO + *hν* → e_CB_^−^ + h_VB_^+^

**Fig. 13:**
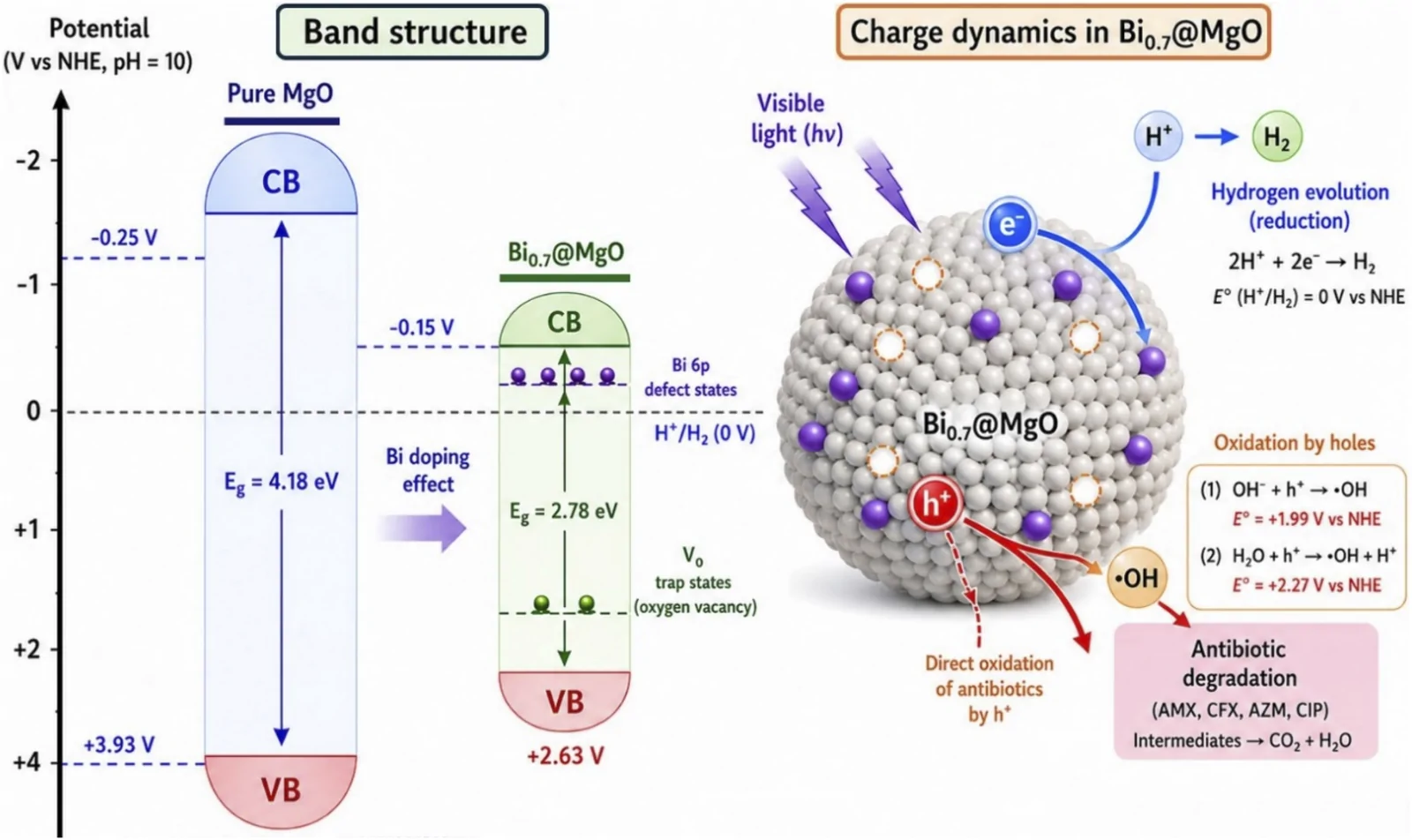
Plausible band structure and photocatalytic charge transfer mechanism of Bi^0^@MgO under simulated solar light irradiation.

Due to the lower Fermi level of metallic bismuth compared to MgO, intimate Bi/MgO contact at the interface drives the migration of electrons until Fermi-level equilibrium is reached. When light is applied, photogenerated electrons migrate preferentially from the MgO conduction band to metallic bismuth, where they accumulate temporarily before participating in proton reduction. The holes remain in the valence band of MgO to conduct oxidation reactions. Oxygen-vacancy defects act as electron traps and facilitate charge transfer at the interface, thereby reducing electron–hole recombination. This explanation is in line with the reduction of the photoluminescence intensity, the reduced charge-transfer resistance from EIS results, and the increased value of transient photocurrent.^37–40^ Because the conduction-band potential of Bi_0.7_@MgO (−0.15 V *vs.* NHE) is more negative than the hydrogen reduction potential (H^+^/H_2_ = 0 V *vs.* NHE), the accumulated electrons possess sufficient thermodynamic driving force to reduce protons into molecular hydrogen:2H^+^ + 2e^−^ → H_2_

Thus, photogenerated electrons are solely responsible for hydrogen evolution. Therefore, the valence-band potential (+2.63 V *vs.* NHE) is considerably more positive than the oxidation potential of hydroxide ions (OH^−^/˙OH = +1.99 V *vs.* NHE) and water (H_2_O/˙OH = +2.27 V *vs.* NHE). Consequently, photogenerated holes readily oxidize surface hydroxyl groups and water molecules to generate highly reactive hydroxyl radicals:OH^−^ + h^+^ → ˙OHH_2_O + h^+^ → ˙OH + H^+^

These hydroxyl radicals, together with the remaining valence-band holes, constitute the dominant oxidative species responsible for the degradation of antibiotic molecules (AMX, CFX, AZM, and CIP). Unlike many photocatalytic systems, the experimentally determined conduction-band potential (−0.15 V *vs.* NHE) is insufficiently negative to directly reduce dissolved oxygen into superoxide radicals (˙O_2_^−^). Therefore, the principal oxidation pathway in Bi_0.7_@MgO proceeds through hole-mediated oxidation and hydroxyl-radical generation, rather than superoxide-radical chemistry. This mechanistic interpretation is fully consistent with the experimentally determined band-edge positions and avoids proposing thermodynamically unfavorable reaction pathways.

Overall, Bi^0^ loading results in (i) narrowing the band gap to improve visible-light absorption, (ii) promoting defect-mediated charge-transfer channels, (iii) suppressing charge recombination, and (iv) optimizing the balance between reduction and oxidation reactions. These synergistic effects enable efficient spatial separation of photogenerated electrons and holes, thereby promoting hydrogen production at the conduction band while driving hydroxyl-radical-assisted antibiotic degradation at the valence band, resulting in the superior bifunctional photocatalytic performance of Bi_0.7_@MgO.

## 4 Conclusion

A sustainable phytomechanochemical strategy was successfully developed to fabricate Bi_*x*_@MgO photocatalysts using clove (*Syzygium aromaticum*) extract as a natural reducing and stabilizing agent combined with solvent-free mechanochemical processing. The approach provides an alternative to conventional solution-based preparation of Bi_*x*_@MgO materials and enables the formation of metallic Bi_*x*_@MgO interfaces without post-synthesis annealing. By varying the Bi loading from 0.2 to 0.7 wt%, systematic changes in the optical and charge-transport properties of MgO were obtained, with Bi0.7@MgO exhibiting the most favorable photocatalytic response. The optimized Bi_0.7_@MgO represented a reduced optical band gap of 2.78 eV along with highly suppressed photoluminescence, reduced charge-transfer resistance, and enhanced transient photocurrent, indicating more efficient photogenerated charge separation and interfacial transport. XPS analysis further confirmed metallic Bi and oxygen-vacancy-related surface species, supporting the contribution of the Bi/MgO interface and defect states to the observed electronic modulation. Under optimized reaction conditions, Bi_0.7_@MgO attained a hydrogen evolution rate of 33.44 mmol g^−1^ h^−1^ under simulated solar irradiation and maintained high activity during repeated cycling. The optimized photocatalyst also exhibited excellent light-driven degradation efficiencies toward amoxicillin, cefixime, azithromycin, and ciprofloxacin, with outstanding stability during repeated photocatalytic cycles.

Mechanistic investigations established a direct relationship between the engineered electronic structure and the bifunctional photocatalytic performance. The excellent agreement among DRS, PL, EIS, transient photocurrent, Mott–Schottky, and LSV analyses confirms that enhanced visible-light harvesting, prolonged charge-carrier lifetime, and accelerated charge transport collectively govern the improved photocatalytic efficiency.

Overall, this study demonstrates that phytomechanochemical-preserved structural engineering is an effective strategy for designing high-performance multifunctional photocatalysts. The realization of efficient solar hydrogen production and antibiotic degradation highlights the potential of Bi_0.7_@MgO as a sustainable photocatalytic platform for integrated renewable energy generation and wastewater remediation. The design strategy presented here provides valuable insights for the development of next-generation multifunctional semiconductor photocatalysts for practical water–energy–environment applications.

## Author contributions

Methodology, I. U., M. K., and M. B. T.; formal analysis, M. A., K. A. T., A. M. A., and R. A.; data curation, I. U., and I. B.; writing – original draft, I. U., M. K., M. N., and M. B. T.; writing – review and editing, M. A., K. A. T., A. M. A., I. B., F. V., R. A., and M. B. T.; supervision, M. B. T.; project administration, F. V., M. B. T., and R. A.; all authors have read and agreed to the published version of the manuscript.

## Conflicts of interest

The authors declare no known competing financial interests or personal relationships that could have influenced the work reported in this paper.

## Supplementary Material

RA-OLF-D6RA08280G-s001

## Data Availability

Data will be available upon reasonable request to the corresponding author. Supplementary information (SI) is available. See DOI: https://doi.org/10.1039/d6ra08280g.

## References

[cit1] Holechek J. L., Geli H. M., Sawalhah M. N., Valdez R. (2022). Sustainability.

[cit2] Johnson N., Liebreich M., Kammen D. M., Ekins P., McKenna R., Staffell I. (2025). Nat. Rev. Clean Technol..

[cit3] Matin Ahmadi S. M., Taghiloo A., Asgharinezhad A. A., Larimi A. (2026). Energy Fuels.

[cit4] Runzhe L., Jingde L., Yu Z., Longde J., Haibin Y., Qingyu C., Zheng Y. (2024). Renewable Sustainable Energy Rev..

[cit5] Boahen E., Owusu L., Adjei-Anim S. O. (2025). Discover Environ..

[cit6] Jayasekara U. G., Hadibarata T., Hindarti D., Kurniawan B., Jusoh M. N. H., Gani P., Tan I. S., Yuniarto A., Rubiyatno, Khamidun M. H. B. (2025). Bioprocess Biosyst. Eng..

[cit7] Sonkar V., Devtalla H., Kumar S. (2025). Environ. Geochem. Health.

[cit8] Eman H. K., Safaa A. M., Noori M. C. S., Nisreen S. A., Issam K. S., Thamer J. M., Talib M. A., Sohrab Z. (2024). Desalin. Water Treat..

[cit9] Ahmed S., Kumar A. (2024). Environ. Sci.: Water Res. Technol..

[cit10] Badar N., Chayed N. F., Roshidah R., Kamarudin N., Kamarulzaman N. (2012). Adv. Mater. Res..

[cit11] Sağlam H. K., İskenderoğlu D., Güldüren M. E., Güney H., Şahin B., Ertuğrul M. (2023). Opt. Mater..

[cit12] Mohd Raub A. A., Bahru R., Mohamed M. A., Latif R., Mohammad Haniff M. A. S., Simarani K., Yunas J. (2024). Nanotechnology.

[cit13] Bishnoi P., Kumar A., Brajpuriya R., Parmar K., Sharma A., Chae K. H., Vij A. (2024). Ceram. Int..

[cit14] Adam F. A., Ghoniem M., Diawara M., Rahali S., Abdulkhair B. Y., Elamin M., Ben Aissa M. A., Seydou M. (2022). RSC Adv..

[cit15] Al-Sharabi A., Sada’a K. S., Al-Osta A., Abd-Shukor R. (2022). Sci. Rep..

[cit16] Irshad Z., Javaid S., Ali O. M., Khan M. S., Alsulami F. M. H., Almasoudi A., Al-Ghamdi A. A., Banbela H. M., Hajjar D., Makki A. A. (2025). ChemistrySelect.

[cit17] Nazir M. K., Taj M. B., Al-Ghamdi A. A., Almasoudi A., Alsulami F. M. H., Banbela H. M., Ali O. M., Ahmed M. M., Khan M. I., Shanableh A. (2025). Catalysts.

[cit18] AlSulami F., Alsabban M. M., Banbela H. M., Zaidi N., Habib S., Hajjar D., Makki A. A., Bibi I., Javed T., Afzal A. (2025). Mater. Adv..

[cit19] Hamza A., Taj M. B., Jahan S., Ejaz S. A., Afzal A., Al Solami A., Zaidi N. (2025). New J. Chem..

[cit20] Mushtaq H., Batool I., Taj M. B., Ali O. M., Alnajeebi A. M., Yahya R., Alelwani W., Alsabban M. M., Kabrah A., Alghamdi S. (2025). New J. Chem..

[cit21] Hamza A., Taj M. B., Alsabban M. M., Altowerqi A. M., Jahan S., Afzal A., Alghamdi S. (2025). RSC Adv..

[cit22] Maseeh I., Anwar F., Aroob S., Javed T., Bibi I., Almasoudi A., Raheel A., Javid M. A., Carabineiro S. A., Taj M. B. (2024). Mater. Adv..

[cit23] Karthikeyan B., Sakthivel A., Kumar P. S., Hussain S. (2024). Surf. Interfaces.

[cit24] Shi M., Su J., Zhang K., Tian Q., Lv X., Shen K., Jia Y., Huang Y., Wang C. (2024). Surf. Interfaces.

[cit25] Tamin S., Dzulkurnain N., Adnan S., Jaafar M., Mohamed N. (2018). J. Sol–Gel Sci. Technol..

[cit26] Ali M. H., Rahman M. A., Hamid A. U., Ahmad H. (2026). RSC Adv..

[cit27] Saha P., Mahiuddin M. (2026). Micro Nano Lett..

[cit28] Kazmi J., Ooi P. C., Goh B. T., Lee M. K., Wee M. M. R., Karim S. S. A., Raza S., Mohamed M. A. (2020). RSC Adv..

[cit29] Matsui M., Kuwata H., Mori D., Imanishi N., Mizuhata M. (2019). Front. Chem..

[cit30] Steffy J. P., Janani B., Mohammed Rafi S., Mohamed E. A., Khan S. S. (2026). J. Water Process Eng..

[cit31] Pan X., Yang M.-Q., Fu X., Zhang N., Xu Y.-J. (2013). Nanoscale.

[cit32] Huang Y., Yu Y., Yu Y., Zhang B. (2020). Sol. RRL.

[cit33] Ashraf M., Guo J., Yan K., Zhang J. (2026). Materials.

[cit34] Wang S., Sun X., Shi J., Zhao R., Zhang B., Lu S., Li P., Li F., Manna L., Zhang Y. (2024). Adv. Mater..

[cit35] Zheng X., Wang X., Feng L., Chen Z., Zhang J., Zhang X., Liu P. (2024). ACS Appl. Mater. Interfaces.

[cit36] Hankin A., Bedoya-Lora F. E., Alexander J. C., Regoutz A., Kelsall G. H. (2019). J. Mater. Chem. A.

[cit37] Schneider J., Matsuoka M., Takeuchi M., Zhang J., Horiuchi Y., Anpo M., Bahnemann D. W. (2014). Chem. Rev..

[cit38] Low J., Yu J., Jaroniec M., Wageh S., Al-Ghamdi A. A. (2017). Adv. Mater..

[cit39] Li J., Li X., Li X. (2025). Discover Mater..

[cit40] Wang Q., Domen K. (2019). Chem. Rev..

