# Supplementary material for: Phytomechanochemical engineering of Bi/MgO interfaces for enhanced solar hydrogen production and multicomponent antibiotic degradation
Source: RSC Adv. 2026 Nov 9. Online ahead of print. doi: 10.1039/d6ra08280g (PMC13746203; doi:10.1039/d6ra08280g)
Supplement: Supplementary file 1 [file RA-OLF-D6RA08280G-s001.pdf]

Supplementary Information (SI) for RSC Advances.
This journal is © The Royal Society of Chemistry

Supporting Information

Characterization techniques

The synthesized samples were subjected to various characterization techniques to determine their structural, surface, and morphological properties. The techniques used include X-ray diffraction (XRD), scanning electron microscopy (SEM), UV-vis spectroscopy, fluorescence, Fourier transform infrared spectroscopy (FTIR). The crystalline structure of synthesized samples were characterized by powder XRD on a Bruker D8 Advance instrument, using Cu-Ka radiation with a wavelength of 1.541 Å. The vibrational frequencies of the prepared composites were determined using FTIR spectroscopy (Tensor 27 FTIR) in the range of 400 to 4000 cm^{-1}. The diffuse reflectance spectroscopy (DRS) measurements were performed using aUV-2550/Shimadzu UV–vis spectrophotometer. The surface and morphological features of the composites were analyzed using SEM (MIRA-III TESCON). Impedance spectroscopy (EIS) using electrochemical techniques was conducted by applying a 10 mV amplitude within a frequency range of 1 MHz to 1.0 Hz while keeping the potential constant at 200 mV (vs.Ag/AgCl). The working electrolyte used in the analysis was 0.1 M sodium sulfate dissolved in water.

Figures

Fig. S1

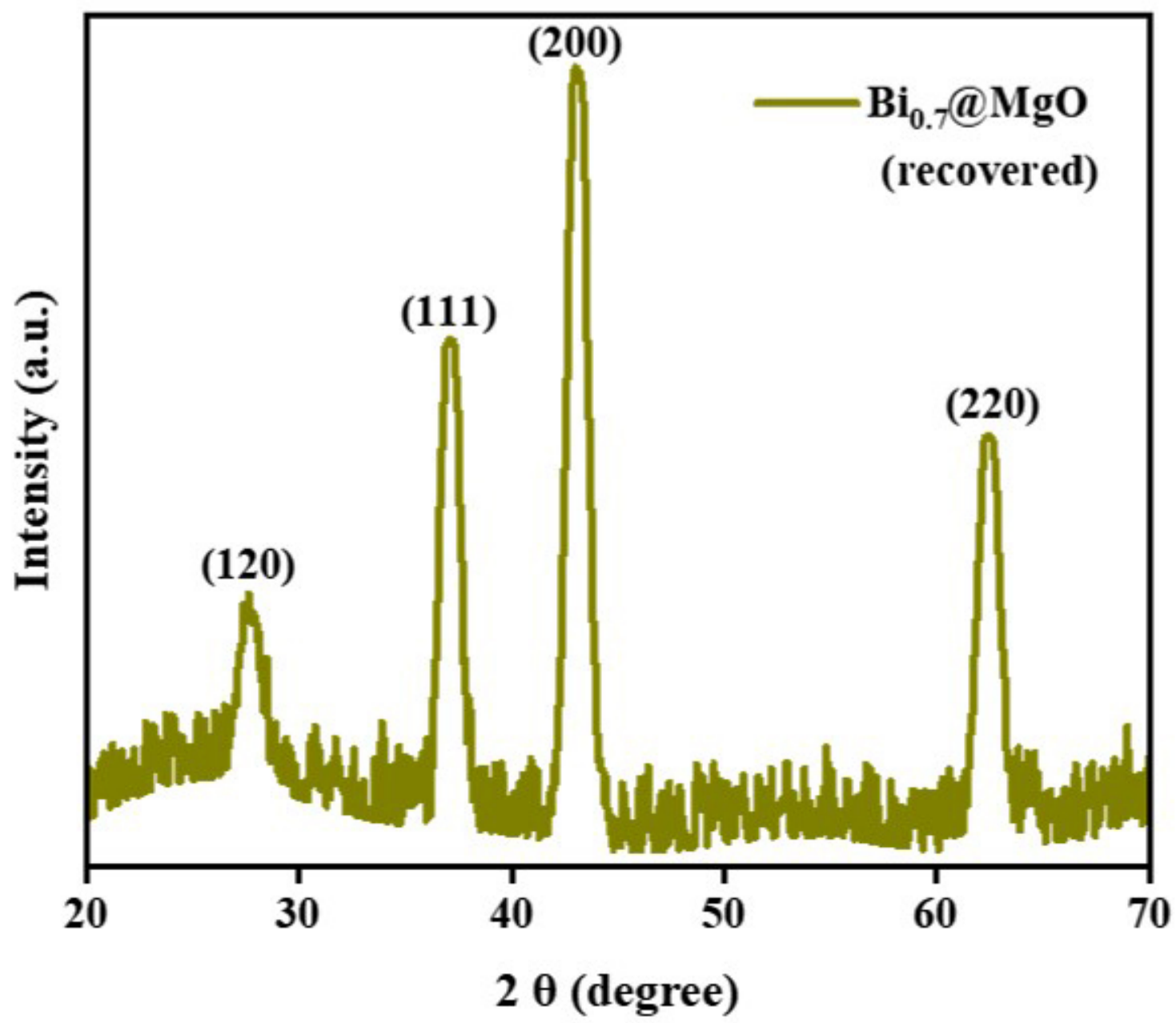

Fig. S1 XRD spectrum of recovered catalyst ($Bi_{0.7}$@MgO) after H_2 production study.

Table

Table S1. Comparison of H_2 evolution performance of MgO-based photocatalytic systems

Photocatalyst	Light source	Reaction conditions	H₂ evolution rate	Co-catalyst	Proposed enhancement mechanism	Ref.
MgO nanocrystals	UV irradiation	Methanol; room temperature; atmospheric pressure	320 μmol/g/h	None	Porous MgO nanocrystals and surface electronic states facilitate methanol photodecomposition and H₂ evolution.	1
MgO/g-C_3N_4	Visible light	Water splitting; 1 wt% MgO/g-C_3N_4	*30.1 μmol/h	None	MgO/g-C_3N_4 heterojunction promotes photogenerated charge transfer and suppresses electron–hole recombination.	2
Ag/MgO	Light-assisted	Formaldehyde/H_2O; O_2^- promoted H₂ evolution	TOF >6600/h	Ag	Interfacial Ag/MgO chemistry and surface-stabilized MgO/Ag–•OOH species promote H_2 evolution.	3
CdS/MgO (commercial MgO)	Blue LED, 450 nm	50 mg catalyst; 200 mL methanol/H₂O (1:1, v/v); 5 h	*3 mmol/g (CdS)/h	None	CdS photoexcitation coupled with MgO; MgO crystal structure and the CdS/MgO interface influence charge separation and H₂ evolution.	4
CdS/MgO (Mg(NO₃)₂-derived MgO)	Blue LED, 450 nm	50 mg catalyst; 200 mL methanol/H_2O (1:1, v/v); 5 h	*8 mmol/g (CdS)/h	None	Precursor-dependent MgO crystallinity, phase composition and CdS/MgO interfacial interactions enhance photocatalytic activity.	4
CdS/MgO (MgCO₃-derived MgO)	Blue LED, 450 nm	50 mg catalyst; 200 mL methanol/H_2O (1:1, v/v); 5 h	*28 mmol/g (CdS)/h	None	Optimized MgO/Mg(OH)₂ composition and MgO crystallite dimensions enhance CdS/MgO interfacial synergy and charge separation.	4
CdS/MgO nanofibers	UV–visible light	Water-splitting/photocatalytic system; 8 wt% CdS	951.79 μmol/g/h	None reported	Oriented MgO nanofibers facilitate charge migration, while the CdS/MgO junction suppresses electron–hole recombination.	5
**$Bi_{0.7}$@MgO**	**Simulated solar light**	**5 mg catalyst; 5 vol% CH_3OH; pH 10**	**33.44 mmol/g/h**	**None**	**Bi modification may regulates defects/oxygen vacancies, modulates the electronic structure, enhances light utilization and promotes photogenerated charge separation.**	**This work**

* The H_2 evolution rates reported for CdS/MgO are normalized to the mass of CdS rather than the total mass of the CdS/MgO composite. The authors used a nominal CdS loading of 5 wt%, while XRD estimated CdS contents of approximately 8, 10 and 13% for the commercial-, nitrate- and carbonate-derived MgO composites, respectively. The photocatalytic tests employed 50 mg catalyst in 200 mL methanol/water (1:1, v/v) under 450 nm blue LEDs.

BET surface area, pore volume and average pore diameter calculation for MgO and $Bi_{0.7}$@MgO

BET surface-area calculation

The BET equation is:

$$\frac{P}{[V(P_o - P)]} = \frac{1}{V_m C} + \frac{C - 1}{V_m C}\left(\frac{P}{P_o}\right)$$

For the calculation, BET linear region P/P_o =0.05-0.30 was used. A linear regression of $y = (P/P_o)/[V(1 - P/P_o]$ versus $x = P/P_o$ gives the slope and intercept as follows;

Parameter	MgO	$Bi_{0.7}$@MgO
BET range (P/P₀)	0.05–0.30	0.05–0.30
R^2	0.9830	0.9713
Monolayer volume, Vₘ	23.94 cm^3 (STP) g⁻¹	30.83 cm^3(STP) g⁻¹
BET constant, C	25.71	22.20
$S_{(BET)}$	104.2 m^2 g⁻¹	134.2 m^2 g⁻¹

Calculation of monolayer volume:

BET regression has slope s and intercept i, then:

$$V_m = 1/(s + i)$$

$$C = 1 + s/i$$

These calculations give V_m = 23.94 cm^3(STP) g⁻¹ for MgO and V_m = 30.83 cm^3(STP) g⁻¹ for $Bi_{0.7}$@MgO.

For surface area:

$$S_{(BET)} = V_m \times N_a \times σ/22414$$

where $N_a = 6.022 \times 10^{23}$ mol⁻¹, σ = 0.162 nm^2 (cross-sectional area of an N_2 molecule), and 22414 cm^3 mol⁻¹ is the molar gas volume at STP.

MgO:

$$S_{(BET)} = 23.94 \times 6.022 \times 10^{23} \times 0.162 \times 10^{-18}/22414 = 104.2\ m^2.g^{-1}$$

$Bi_{0.7}$@MgO:

$S_{(BET)} = 30.83 \times 6.022 \times 10^{23} \times 0.162 \times 10^{-18}/22414 = 134.2\ m^2.g^{-1}$

Total pore-volume calculation

The N_2 uptake near $P/P_0 = 0.99$ was used as the total adsorbed volume. The values are approximately 94.12 cm^3(STP) g^{-1} for MgO and 125.06 cm^3(STP) g^{-1} for $Bi_{0.7}$@MgO.

Conversion from gas volume at STP to liquid-N_2 pore volume:

$$V_p = V_{(STP)} \times 34.65/22414$$

MgO:

$$V_p = 94.12 \times 34.65/22414 = 0.146\ cm^3\ g^{-1}$$

$Bi_{0.7}$@MgO:

$$V_p = 125.06 \times 34.65/22414 = 0.193\ cm^3\ g^{-1}$$

Here, 34.65 $cm^3\ mol^{-1}$ is the approximate molar volume of liquid N_2

Average pore diameter

Assuming cylindrical pores, the average pore diameter can be estimated from the surface area and pore volume using:

$$D_p(nm) = 4000\ V_p/S_{(BET)}$$

MgO:

$$D_p = 4000 \times 0.146/104.2 = 5.59\ nm$$

$Bi_{0.7}$@MgO:

$$D_p = 4000 \times 0.193/134.2 = 5.76\ nm$$

References

1. Z. Liu, Z. Yin, C. Cox, M. Bosman, X. Qian, N. Li, H. Zhao, Y. Du, J. Li and D. G. Nocera, *Science advances*, 2016, **2**, e1501425.
2. N. Mao and J.-X. Jiang, *Applied Surface Science*, 2019, **476**, 144-150.
3. R. Li, X. Zhu, X. Yan, H. Kobayashi, S. Yoshida, W. Chen, L. Du, K. Qian, B. Wu and S. Zou, *Acs Catalysis*, 2017, **7**, 1478-1484.
4. A. G. Romero-Villegas, F. Tzompantzi, S. Ramírez-Rave, A. Hernández-Gordillo, J. C. Ruiz, G. Morales-Mendoza and R. Gómez, *Journal of Materials Science: Materials in Engineering*, 2025, **20**, 74.
5. M. Huang, L. Xing and X. Zheng, *International Journal of Hydrogen Energy*, 2026, **214**, 153829.
